# *miR-137* targets *Myc* to regulate growth during eye development

**DOI:** 10.1242/dev.204373

**Published:** 2025-07-16

**Authors:** Radhika Padma, Manivannan Subramanian, Anuradha Venkatakrishnan Chimata, Arushi Rai, Sunanda Yogi, Anjali Sangeeth, Madhuri Kango-Singh, Amit Singh

**Affiliations:** ^1^ Department of Biology, University of Dayton, Dayton, OH 45469, USA; ^2^ Premedical Program, University of Dayton, Dayton, OH 45469, USA; ^3^ Center for Genomic Advocacy (TCGA), Indiana State University, Terre Haute, IN 47809, USA

**Keywords:** *Drosophila*, Eye development, Retinal determination, Retina, miRNA, miR-137, *Myc*, Cell death, Cell proliferation

## Abstract

During development, regulation of gene expression is key to cellular homeostasis. Gene expression regulation by non-coding RNAs involves the prevention of mRNA accumulation or the inhibition of translation of their target gene. In a forward-genetic screen to identify the microRNA involved in the growth and patterning of the *Drosophila* eye, we identified the highly conserved *miR-137*. Gain of function of *miR-137* results in a reduced-eye phenotype by downregulating retinal determination and differentiation markers, and by upregulating negative regulators of eye development, such as Wingless (Wg) and Homothorax (Hth). Loss of function of *miR-137* results in an enlarged-eye phenotype. Using bioinformatics and genetic approaches, we identified the oncogene *Myc* as the target of *miR-13*7. Gain of function of *Myc* can rescue the reduced-eye phenotype of *miR-137* gain of function, and vice versa. We tested the role of *miR-137* in regulating *Myc* levels in the *Ras^V12^;scrib^RNAi^*, a tumor model of oncogenic cooperation that results in neoplastic tumors. Gain of function of *miR-137* in the *Ras^V12^;scrib^RNAi^* background significantly reduced tumor phenotype as well as *Myc* levels in the eye. Our studies highlight *miR-137* as a post-transcriptional regulator of *Myc* and a promising therapeutic target for diseases associated with *Myc* accumulation.

## Teaser

**Summary:** Identification and characterization of *miR-137* as a post-transcriptional regulator of *Myc* that controls growth, offering insights into diseases such as neurodegenerative disorders and cancer, which are linked to Myc accumulation.

## INTRODUCTION

During development, complex cell biological processes such as cell fate specification, cell proliferation, patterning, growth and cell death are strictly regulated by precise spatial and temporal regulation of the expression of genes. Regulation of gene expression occurs through multiple mechanisms, like regulation of transcription mediated by transcription factors, transcriptional pausing mechanisms or post-transcriptional mechanisms involving microRNAs (miRNAs) (Ben-Hamo and Efroni, 2015; Chimata et al., 2023; Li and Gilmour, 2011; Shang et al., 2023). Among these mechanisms, the developmental roles of miRNAs are beginning to emerge. miRNAs were initially discovered in *Caenorhabditis elegans* as a new mechanism of temporal regulation of genes and cell lineages (Lee et al., 1993). miRNAs are a class of small non-coding RNAs averaging 22 nucleotides in length that are encoded in the genome, transcribed into primary miRNAs, processed into precursors in the nucleus, and finally mature into miRNA in the cytoplasm (Bartel, 2004). miRNAs interact with 3′UTR of the target gene mRNAs to generate double-stranded RNA to induce target mRNA degradation, translational repression, mRNA deadenylation and decapping (Shang et al., 2023; Vishnoi and Rani, 2023). miRNAs confer specificity to the RNA-induced silencing complex (RISC) through partial sequence complementarity with specific mRNA targets (Ben-Hamo and Efroni, 2015; Shang et al., 2023). Recruitment of miRNA and the RISC complex mostly results in repression of the target mRNA by an increase in turnover and/or translational inhibition. Thus, miRNAs function in fine-tuning the activity of the target gene(s), thereby enhancing the robustness of gene regulatory networks (Ebert and Sharp, 2012). Recent findings indicate that miRNAs play a crucial role in development, differentiation, the modulation of multiple signaling pathways, cell cycle regulation, the stress response, behavior, hormone action and longevity (Ambros and Ruvkun, 2018; Ben-Hamo and Efroni, 2015; Frédérick and Simard, 2022). In humans, aberrant expression of miRNA(s) is implicated in cancer, and in cardiovascular and metabolic disorders; it is also used as a molecular marker for these conditions (Deshpande et al., 2024; Kariuki et al., 2023; Vaghf et al., 2022).

To study miRNA functions, *Drosophila* has proved to be an effective model system with its array of genetic tools and high degree of gene conservation (Bier, 2005; Perry et al., 2017; Singh and Irvine, 2012). The *Drosophila* eye model can be a suitable model for identifying and characterizing miRNA functions, as it has been extensively used to investigate cell fate specification, growth, cell-cell communication, cell survival and cell death mechanisms during organogenesis (Singh and Choi, 2003; Singh and Irvine, 2012; Singh et al., 2005b; Verghese et al., 2012; Warren and Kumar, 2023; Wolff and Ready, 1993). The power of the *Drosophila* eye as a model lies in the conservation of genetic machinery at the structural as well as the functional level. Furthermore, the roles of miRNAs in different cell types of the developing retina are conserved across species (Reh and Hindges, 2018).

*Drosophila* eye develops from a monolayer epithelium housed inside the *Drosophila* larva, called an eye imaginal disc. The larval eye imaginal disc gives rise to the adult compound eye, antenna and head cuticle (Cohen, 1993; Gogia et al., 2020a; Mishra and Sprecher, 2020; Tare et al., 2013). During the late second instar stage, the process of retinal differentiation begins as a synchronous wave, called the morphogenetic furrow (MF), at the posterior margin of the eye imaginal disc and moves toward the anterior end (Kumar, 2020; Ready et al., 1976). The cells posterior to the MF differentiate into photoreceptor neurons (Kumar, 2020; Warren and Kumar, 2023), controlled by the highly conserved retinal determination and differentiation (RD) gene network. The RD gene network comprises PAX-6 homolog *eyeless* (*ey*)*, twin of eyeless* (*toy*)*, eye gone* (*eyg*), *twin of eyegone* (*toe*), *eyes absent* (*eya*), *sine oculis* (*so*) and *dachshund* (*dac*), which are necessary and sufficient for normal eye development (Bui et al., 2000; Gogia et al., 2020a; Halder et al., 1998; Jang et al., 2003; Kango-Singh et al., 2003). In the first instar larva, *ey*, the master regulator of eye fate, is expressed in the entire eye disc. However, when differentiation begins, *ey* expression is downregulated in the cells posterior to the MF, and expression of downstream genes *eya*, *so* and *dac* is activated to promote retinal differentiation (Bessa et al., 2002; Kumar, 2010; Singh et al., 2002). At the MF, Eya, Dac and So activate *atonal* (*ato*), which is required for the initiation of R8 differentiation (Jarman et al., 1994; Tanaka-Matakatsu and Du, 2008). The uniform spacing between photoreceptor clusters is maintained by the fibrinogen-related secreted protein Scabrous (Sca) (Baker et al., 1990). Prospero (Pros) is a marker for R7 photoreceptor fate and cone cells in the eye (Hayashi et al., 2008).

The differentiation along the MF is also guided by signaling molecules, such as Hedgehog (Hh) and Decapentaplegic (Dpp) (Domínguez and Hafen, 1997; Kumar, 2020; Sarkar et al., 2018). The *dpp* expression moves dynamically with the MF, thus serving as an excellent marker for MF progression (Kumar, 2020; Sarkar et al., 2018; Warren and Kumar, 2023). Dpp promotes MF progression by downregulating *wingless* (*wg*), a ligand for the evolutionarily conserved Wg/WNT signaling pathway. In the developing eye, *wg* is expressed along the antero-lateral margins of the third-instar eye imaginal disc. Wg blocks MF progression by negatively regulating photoreceptor differentiation and thereby determines the eye versus head fate (Ma and Moses, 1995; Puli et al., 2024; Tare et al., 2013; Treisman and Rubin, 1995; Won et al., 2015). Furthermore, Wg regulates the expression of *homothorax* (*hth*), which encodes a TALE (three amino-acid loop extension) type homeodomain protein, to suppress eye fate. During larval eye development, Hth expression evolves and retracts anteriorly, ahead of the MF, in the late second instar. In the third instar larval eye-antennal imaginal disc, Hth expression is in the anterior region of the eye disc (Bessa et al., 2002; Pai et al., 1998; Singh et al., 2004, 2011, 2012). Along with the control of differentiation, the overall growth of the eye is also tightly regulated by growth regulatory pathways such as Hippo and PI3K/TOR. These pathways control overall organismal growth by controlling cell proliferation, cell size and cell death (Brennecke et al., 2003; Verghese et al., 2012; Wittkorn et al., 2015). Thus, eye differentiation is orchestrated by the interactions of the RD genes and signaling pathways.

Ectopic Wg can suppress eye fate through activation of caspase-dependent cell death (Singh et al., 2006). The initiator caspase Dronc is activated by upstream genes *reaper* (*rpr*)*, head involution defective* (*hid*) and *grim* (also known as RHG) (Chen et al., 1996; Grether et al., 1995; White et al., 1994). Caspases are essential for maintaining cellular homeostasis by facilitating a programmed cell death process called apoptosis. It involves the formation of the apoptosome, which is a complex of adaptor protein(s) that triggers the activation of the initiator caspase leading to apoptosis. Another mode of cellular homeostasis, autophagy, represents a unique mechanism of cell death that involves the removal of damaged or surplus cytoplasmic components through a catabolic process mediated by lysosomal degradation. Autophagy is genetically regulated by evolutionarily conserved genes involved in autophagolysosomal degradation. In *Drosophila,* there are 20 highly conserved autophagy-related genes (Atg) identified. Among these, the Atg8 gene family (Atg8a and Atg8b) has been extensively studied, with its members serving as reliable biomarkers for autophagy (Chimata et al., 2023; Larkin et al., 2021; Shpilka et al., 2011).

We performed a forward-genetic screen to uncover miRNAs involved in eye development. Specifically, we screened for changes in eye size upon overexpression of the miRNAs in the developing eye using the well-established GAL4-UAS system (Brand and Perrimon, 1993; Deshpande et al., 2024). Here, we report the identification of *miR-137* in a forward-genetic screen for modifiers of eye size. Gain of function of *miR-137* results in reduced-eye phenotype by affecting the RD gene network and activating the expression of negative regulators such as Wg and Hth. The gain-of-function phenotype of *miR-137* has no domain constraint. Loss of function of *miR-137* results in an eye-enlargement phenotype. We identified *Myc* (also known as d*Myc* and c*Myc*), a *Drosophila* homolog of the vertebrate Myc oncogene (Gallant et al., 1996; Johnston et al., 1999), as the target of *miR-137* in the eye, where it regulates cell homeostasis to determine the size of the eye field. *Myc* is a transcription factor and a proto-oncogene that plays a dual role during development. *Myc* expression is closely linked to cell-cycle progression in normal tissues, whereas uncontrolled *Myc* expression is a hallmark of hyperproliferation observed in many cancers. Interestingly, despite its role in promoting cell proliferation, *Myc* also serves as a potent inducer of apoptosis. Thus, *Myc* regulates growth via proliferation, cell death and metabolism during development. We employed the *Ras^V12^; scrib^RNAi^* tumor model for oncogenic cooperation to demonstrate that the gain of function of *miR-137* can significantly rescue the neoplastic overgrowth phenotype(s) by downregulating *Myc* levels. Here, we present a previously unreported function for highly conserved *miR-137* in regulating tissue homeostasis and growth by targeting *Myc* during eye development.

## RESULTS

### Gain of function of *miR*-*137* results in reduced-eye phenotype

**Fig. 1.**
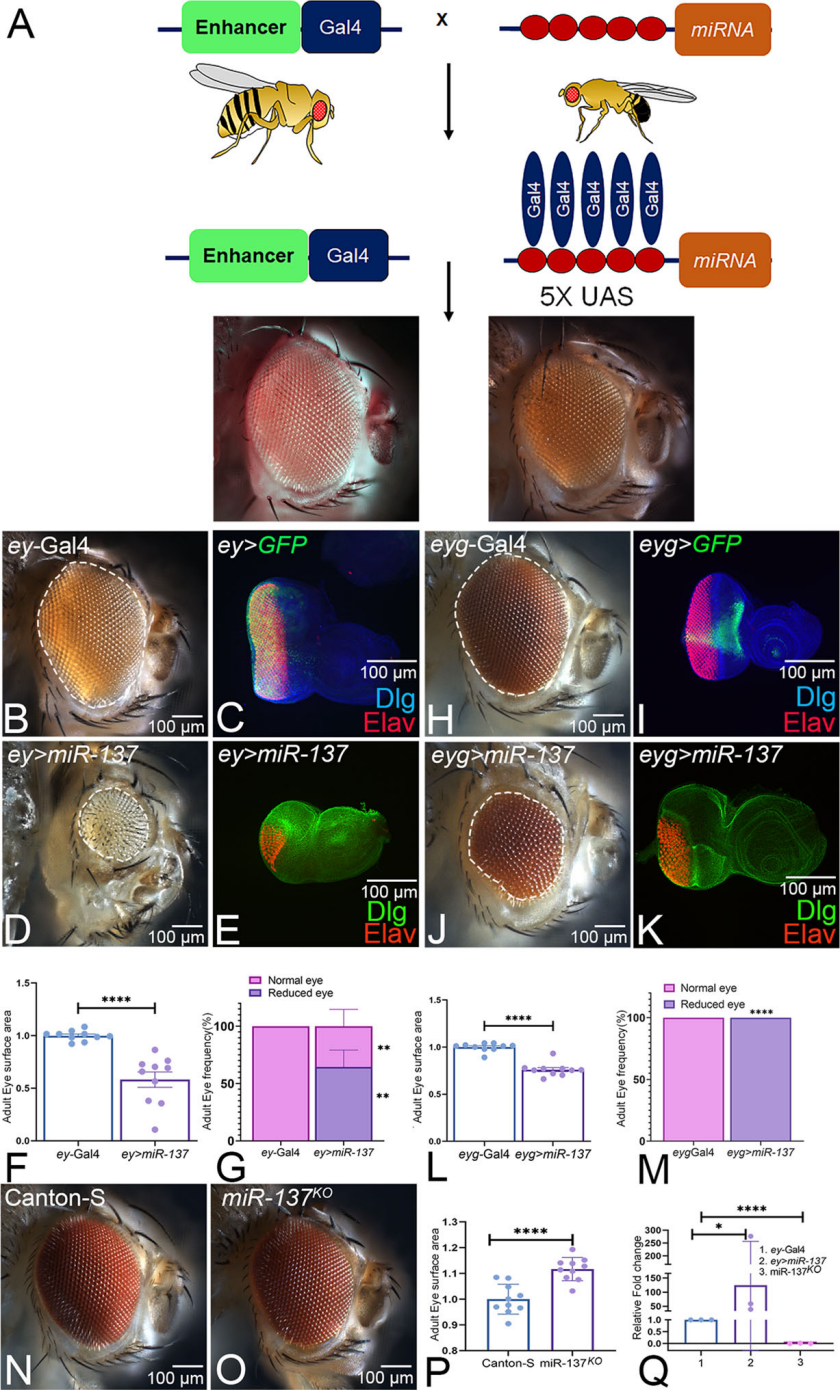
**Screening for miRNA involved in eye development results in the identification of *miR-137*.** (A) The screening strategy to identify miRNA(s) involved in eye development. In the developing eye, individual miRNA(s) are misexpressed using the Gal4/UAS Target system, and their effect on eye phenotype is assayed. (B) The adult eye of an *ey*-Gal4 control, to which misexpressed miRNA(s) phenotypes were compared. (C) The domain of expression (UAS GFP, green) of *ey*-GAL4 in the third-instar larval eye-antennal imaginal disc stained for Discs large (Dlg, blue), to mark cellular outlines, and Elav (red), to mark photoreceptor neurons. (D,E) Gain of function of *miR-137* in the developing eye using *ey*-Gal4 driver (*ey>miR-137*) results in the reduced-eye phenotype, as seen in (D) adults and (E) larval eye imaginal disc. (F,L,P) Quantification of adult eye surface area (μm^2^) using Fiji/ImageJ software with statistical analysis of normalized adult eye surface area (150 pixels/inch) carried out using an unpaired Student's *t*-test. The analyzed area is indicated with white dotted lines for denoted genotypes (B,D,H,J) (*n*=10). (G,M) Adult eye phenotype frequency of indicated genotypes (*n*=200, three replicates). Analysis was carried out using a two-way ANOVA with Sidak's multiple comparison test. (H) Adult eye of the *eyg*-Gal4 driver serves as a control. (I) The third-instar larval eye-antennal imaginal disc showing *eyg*-Gal4 expression (UAS-GFP, green). (J,K) *eyg>miR-137* exhibits the reduced-eye phenotype in (J) adult and (K) larval eye imaginal disc. (N,O) Adult eyes of Canton-S control (N) and *miR-137^KO^* homozygous (O) flies, which exhibit an enlarged eye phenotype. (Q) The log2 fold-change values of *miR-137* normalized to 2S-rRNA in *ey*-Gal4, *ey>miR-137* and *miR-137^KO^*. Pairwise comparisons were made with an unpaired *t*-test. *miR-137* is expressed in the developing eye. All graphs were plotted using GraphPad Prism 8.3.1. Data are mean±s.e.m. Statistical significance is indicated in each graph: ****P<0.0001; **P<0.01; *P<0.05; ns, non-significant. Orientation of all the imaginal discs is posterior to the left and dorsal upwards. All eye-antennal imaginal discs were captured at 20× magnification and adult eyes at 10× magnification, unless specified otherwise. Scale bars: 100 µm.

We performed a forward genetic screen in *Drosophila* to look for miRNAs involved in eye development, where we individually express miRNA transgene(s) in the developing eye (Fig. 1A). We used the *ey*-Gal4, which drives the expression of GFP reporter (*ey*-*GAL4*>*UAS-GFP*, *ey>GFP*) in the entire eye field (Fig. 1C), and assayed its effect on the eye phenotype in the F1 progeny (Fig. 1A) (Singh et al., 2005a). Among various miRNA(s) misexpressed in the developing eye field (Fig. 1C), we found that, compared to the control *ey>GFP* (Fig. 1B,C), the gain of function of *miR-137* reduces the eye size significantly both in the adult and the larval eye imaginal disc (Fig. 1D,E). Furthermore, statistical analysis of the eye-surface area of *ey>miR-137* flies showed a significant reduction (42%) compared to the adult eyes of *ey*-Gal4 control (Fig. 1F). In addition, the frequency of reduced-eye phenotype of adult eye in *ey>miR-137* was significantly higher at 64.5% (*n*=387/600) when compared to the *ey*-Gal4 control, where 100% of flies showed normal wild-type adult eye (Fig. 1G). We used *eyegone* (*eyg*)-GAL4, another Gal4, to confirm the phenotypic defects in the eye caused by the gain of function of *miR-137*. *eyg-*Gal4 drives the GFP reporter expression (*eyg-*Gal4>UAS-GFP, *eyg*>GFP) in an antero-dorsal stripe in the mid-late third-instar larval eye disc (Fig. 1I), a pattern that closely mimics *eyg* gene expression (Jang et al., 2003). The control *eyg-*Gal4 exhibits a wild-type eye phenotype in the imaginal discs and adults (Fig. 1H,I). Targeted expression of *miR-137* by *eyg*-Gal4 (*eyg>miR137*) resulted in a reduced-eye phenotype, as seen in the adult as well as in the eye imaginal disc (Fig. 1J,K). Furthermore, statistical analysis of the eye-surface area and the frequency of reduced-eye phenotype of *eyg>miR-137* adult eye showed a significant reduction (24%) of eye-surface area and a significant increase in reduced-eye frequency, when compared to the *eyg*-Gal4 control (Fig. 1L,M). These results suggest that gain of function of *miR-137* results in reduced-eye phenotype.

To understand the role of *miR-137* in eye development, we assayed the *miR-137* loss-of-function phenotype using three different alleles, i.e. *miR-137*^KO^ (Fig. 1O), *miR-137CR* and *ey>miR-137* sponge (Fig. S1). We found a significant increase in the eye size of *miR-137^KO^* (Fig. 1O,P) when compared to Canton-S (Fig. 1N,P). In addition, homozygous *miR-137CR* and *ey>miR-137* sponge exhibits increased eye size in comparison to Canton-S and *ey*-Gal4 (Fig. S1). Additionally, to validate the expression levels of *miR-137* during eye development, we performed qRT-PCR and found that *miR-137* is expressed in the control larval eye disc (Fig. 1Q). These *miR-137* levels increased significantly in the gain-of-function background of *ey>miR-137* and were non-detectable in *miR-137^KO^* (Fig. 1Q). Our results show that *miR-137* is expressed in the developing eye field, and its loss of function leads to the enlarged eye phenotype. It is important to determine the effect of the reduced-eye phenotype of *miR-137* misexpression on the retinal determination and differentiation gene cascade.

### *miR-137* suppresses retinal determination and differentiation

**Fig. 2.**
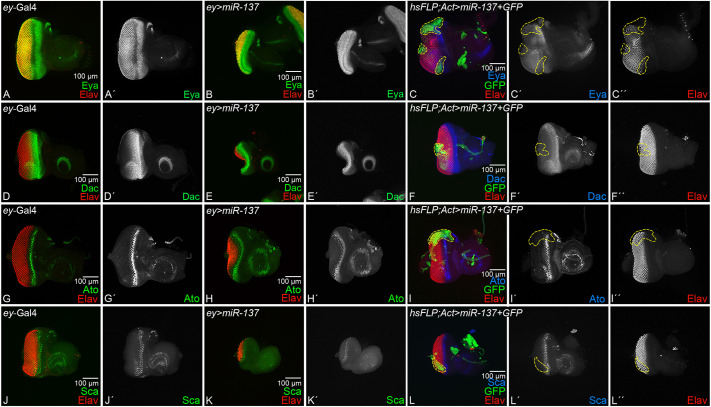
**Gain of function of *miR-13*7 downregulates retinal determination and retinal differentiation genes.** (A-F″) Expression of the retinal determination genes (A-C″) Eya (green) and (D-F″) Dac in eye-antennal imaginal disc of (A,A′,D,D′) *ey*-Gal4, (B,B′,E,E′) *ey>miR-137* and (C-C″,F-F″) hsFLP-out clones of *miR-137*. (A′,B′,C′,D′,E′,F′) Single channel images for (A′,B′,C′) Eya and (D′,E′,F′) Dac. Elav (red) marks the retinal neuron. (B,B′) Eya and (E,E′) Dac expression is downregulated posterior to the MF in the *ey>miR-137* background, which exhibits a reduced-eye phenotype. FLP-out clones showed abnormal spacing between neighboring photoreceptors and exhibited downregulation of (C,C′) Eya and (F,F′) Dac. (C″,F″) Elav indicates where retinal neurons are affected. (G-L″) Expression of a retinal differentiation genes (G-I″) Ato (green) and (J-L″) Sca (green) in eye-antennal imaginal disc of (G,G′,J,J′) *ey*-Gal4 control, (H,H′,K,K′) *ey>miR-137* and (I,I′,I″,L,L′,L″) FLP-out clones of *miR-137*. (G′,H′,I′,J′,K′,L′) Single channel images for (G′,H′,I′) Ato and (J′,K′,L′) Sca. (H,H′) Ato and (K,K′) Sca expression is downregulated in MF in the *ey>miR-137* background and a similar observation was seen in (I,I′,L,L′) hsFLP-out clones. All the imaginal discs are oriented posterior to the left and dorsal upwards. All eye-antennal imaginal discs were captured at 20× magnification, unless specified otherwise. Scale bars: 100 µm.

We examined the retinal determination (RD) gene expression in the gain-of-function background of *miR-137* using *ey-*Gal4 driver (*ey>miR-137*) and random FLP-out clones using heat-shock Flippase *(hsFLP; Actin>y+>GAL4/UAS-miR-137)* in the developing eye imaginal discs. There is a significant reduction in the size of the eye field in *ey>miR-137* background. We tested the expression of the two core RD genes, *eyes absent* (*eya*) and *dacshund* (*dac*) in developing eye imaginal discs. The *eya* is expressed in a stripe of retinal precursor cells ahead of the advancing morphogenetic furrow (MF), as well as in the differentiating photoreceptor neurons, cones and pigment cells of the neural retina behind the MF (Bonini et al., 1993), (Fig. 2A,A′). Furthermore, *eya,* along with *sine oculis* (*so*), activates expression of the downstream RD gene *dac* in two different domains that span anteriorly and posteriorly to the MF (Chen et al., 1997), (Fig. 2D,D′). Gain of function of *miR-137* in the developing eye (*ey>miR-137)*, showed that Eya (Fig. 2B,B′) and Dac (Fig. 2E,E′) expression domains are significantly reduced posteriorly to the MF, as compared to the control (Fig. 2A,A′,D,D′). To address the issue of domain constraint, we generated heat-shock Flippase-mediated random FLP-out clones of *miR-137* . We found downregulation of expression of the retinal fate marker Elav as well as Eya and Dac expression downregulation close to the clonal boundary (Fig. 2C,C′,F,F′). Additionally, the arrangement of photoreceptors was disrupted and there was an increase in the distance between photoreceptors (Fig. 2C″,F″).

Furthermore, in the *ey>miR-137* background, we examined the expression of retinal differentiation markers such as Atonal (Ato) (Fig. 2G,G′), Scabrous (Sca) (Fig. 2J,J′) and Prospero (Pros) (Fig. S2) (Baker et al., 1990; Jarman et al., 1994; Mlodzik et al., 1990). Compared to the *ey*-Gal4 control (Fig. 2G,G′,J,J′), gain of function of *miR-137* in the developing eye (*ey>miR-137*) showed reduced expression of Ato (Fig. 2H,H′), Sca (Fig. 2K,K′) and Pros (Fig. S2). In FLP-out clones, the Ato and Sca expression domains were affected. Ato was downregulated in the clone near dorsal eye margin (Fig. 2I,I′) and Sca also exhibited downregulation in the clone (Fig. 2L,L′), and there was disruption of photoreceptor arrangement and spacing (Fig. 2I″,L″). These observations suggest that the gain of function of *miR-137* downregulates the retinal determination and differentiation markers, and FLP-out clones show evidence of impaired photoreceptor arrangement. Our results show that the entire eye field is reduced (evident in Fig. 2B,E,H,K) compared to the controls (Fig. 2A,D,G,J). Taken together, the reduced-eye phenotype of *miR-137* overexpression showed reduced retinal determination and differentiation markers.

### *miR-137* blocks MF progression by activation of negative regulators of the eye

**Fig. 3.**
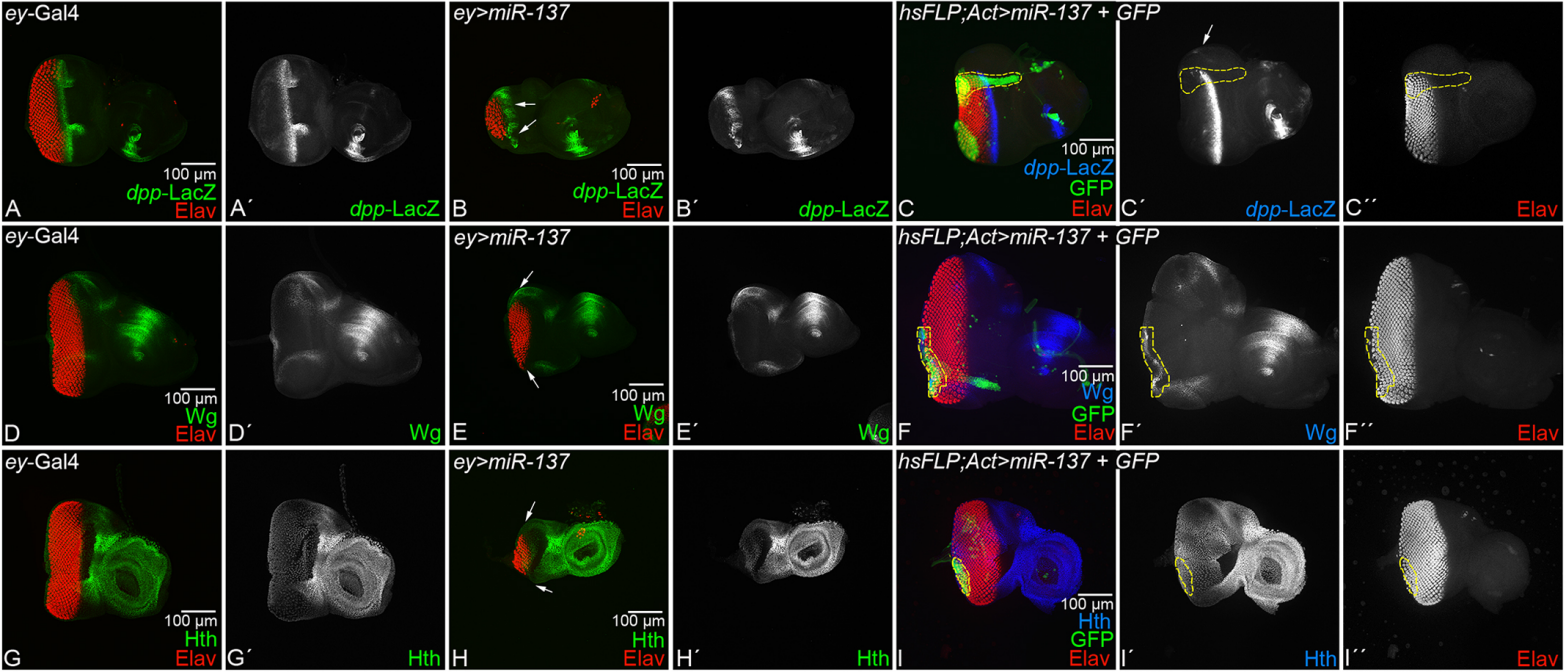
**Gain of function of *miR-137* blocks morphogenetic furrow progression and ectopically induces the negative regulators of eye fate.** (A-C′) Eye-antennal imaginal discs stained for the *dpp-*LacZ reporter (green), which marks the morphogenetic furrow (MF) in the (A,A′) *ey*-Gal4 (control) and (B,B′) *ey>miR-137* background, and in (C,C′) heat shock FLP-out clones of *miR-137* in the developing eye imaginal disc. Elav (red) marks the retinal neuron. (A′,B′,C′) Single channel images for *dpp-*lacZ expression. *dpp*-lacZ is significantly downregulated in *ey>miR-137* background (marked by arrow in C′) when compared to the control and in FLP-out clones. *dpp*-LacZ is downregulated in the border of the clone and does not extend to the dorsal margin (marked by arrows in B,D,H). (D-I″) Eye-antennal imaginal disc of third-instar larvae stained for (D-F″) Wg and (G-I″) Hth. (D-F″) Wg (green), a negative regulator of MF progression, is ectopically induced in (E,E′) the *ey>miR-137* background and in (F,F′) the FLP-out clone of *miR-137* (outlined in yellow) in comparison to the control (D,D′) *ey*-Gal4. (G-I″) Hth (green), a head fate marker and negative regulator of eye fate, is ectopically induced in (H,H′) the *ey>miR-137* background and in (I-I″) the FLP-out clone of *miR-137* (outlined in yellow) in comparison to the control (G,G′) *ey*-Gal4. (D′,E′,F′,G′,H′,I′) Single channel images for (D′,E′,F′) Wg and (G′,H′,I′) Hth. All the imaginal discs are oriented posterior to the left and dorsal upwards. All eye-antennal imaginal discs were captured at 20× magnification, unless specified otherwise. Scale bars: 100 µm.

We investigated the effect of gain of function of *miR-137* (*ey>miR-137*) on MF progression by using a transcriptional reporter of *dpp* as a MF marker (Blackman et al., 1991). In the control eye imaginal discs, *dpp-*lacZ marks a thin stripe that overlays the apical constrictions caused by the MF cells. Compared to the control, in which *dpp*-lacZ expression marks the MF (Fig. 3A,A′), *dpp-lacZ* expression in the *ey>miR-137* background is significantly downregulated (Fig. 3B,B′, arrows). A similar downregulation of *dpp*-lacZ reporter was seen in random FLP-out clones of *miR-137* near the dorsal eye margin in the developing eye (Fig. 3C-C″, arrow). All the random clone boundaries are marked by a yellow dotted line.

In the developing eye, Wg serves as a negative regulator of eye fate and is known to block MF progression (Kumar, 2011; Ma and Moses, 1995; Swarup and Verheyen, 2012; Treisman and Rubin, 1995). Therefore, we tested if the gain of function of *miR-137* (*ey>miR-137*) affects Wg expression*.* In the third-instar larva eye disc, Wg is expressed on the antero-lateral margins of the control eye imaginal disc (Fig. 3D,D′). In the *ey>miR-137* background, which exhibits a reduced-eye phenotype, there is a strong induction of Wg on the margins (Fig. 3E,E′, arrows). Random FLP-out clones of *miR-137* on the posterior margin of the developing eye disc exhibit loss of photoreceptor cells along with the ectopic induction of Wg expression (Fig. 3F-F″). Wg is known to activate Hth – a negative regulator of eye development (Bessa et al., 2002; Pai et al., 1998; Singh et al., 2002). In the wild-type eye disc, *hth* is expressed in the peripodial membrane cells, and in a band of retinal precursor cells that are anterior to the MF (Fig. 3G,G′). We found an ectopic induction of Hth in the *ey>miR-137* reduced-eye field compared to the control (Fig. 3H,H′, arrows). We also tested Hth expression in random FLP-out clones of *miR-137* where the clone boundary is marked by yellow dotted line. We found that Hth is induced on the posterior margin, accompanied by a loss of photoreceptor cells (Fig. 3I-I″). We also tested the expression of F-actin, which also marks MF in the eye (Benlali et al., 2000). We found that in gain of function of *miR-137*, F-actin levels were downregulated (Fig. S2). Overall, these data suggest that *miR-137* plays a role in suppressing retinal fate by promoting the expression of negative regulators of eye, Wg and Hth, and by downregulating the MF marker *dpp* in the developing eye.

### Gain of function of *miR-137* triggers cell death

**Fig. 4.**
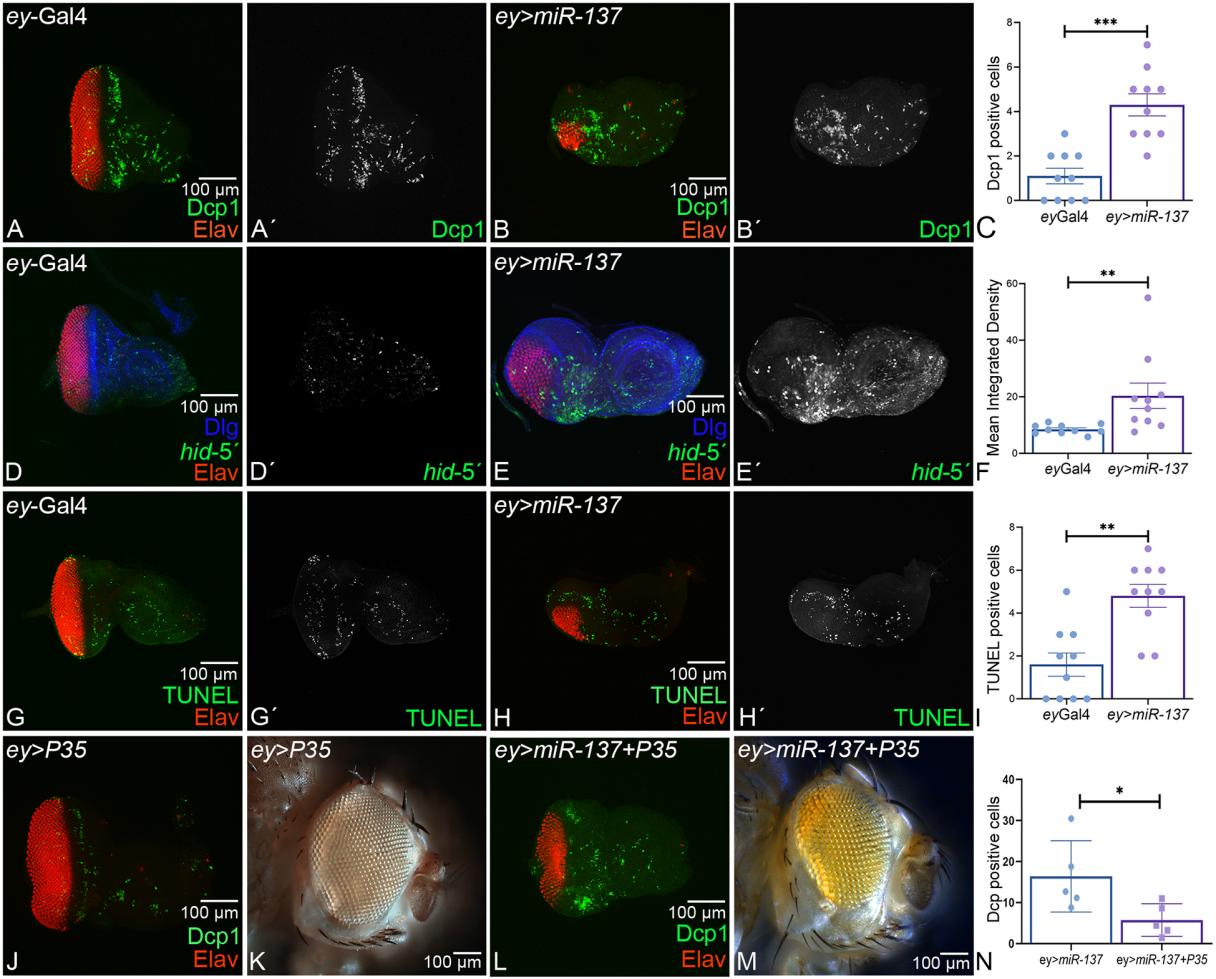
**Gain of function of *miR-137* triggers cell death in the developing eye.** (A-B′,D-E′,G-H′) Eye-antennal imaginal discs of third-instar larvae stained for Elav (red), (A-B′) Dcp1 (green, a marker for apoptotic cells), (D-E′) *hid5′-WT-GFP* (green, a proapoptotic gene, *hid* reporter) and (G-H′) TUNEL (green, a marker for dying cells nuclei) in (A,A′,D,D′,G,G′) *ey*-Gal4 and (B,B′,E,E′,H,H′) *ey>miR-137* background. Single channel images for (A′,B′) Dcp-1, (D′,E′) *hid5′-WT-GFP* and (G′,H′) TUNEL alone. (C,F,I) Statistical analysis showed that ectopic expression of *miR-137* (*ey>miR-137*) exhibits a significant increase in (C) Dcp-1, (F) *hid5′-WT-GFP* and (I) TUNEL-positive nuclei as compared to the *ey*-Gal4 controls as shown in the graphs. The signal intensity was calculated in three regions of interest per disc within a sample of *n*=5 for each genotype. (J-M) Ectopic expression of baculovirus P35, which blocks cell death, in (J,K) *ey*-Gal4 (*ey>P35*, control) and (L,M) *ey>miR-137 (ey>miR-137+P35)* background. P35 rescues the *ey>miR-137* reduced-eye phenotype, as seen in (L) eye imaginal disc and (M) adult eye. (N) Statistical analysis showed a decrease in Dcp1-positive cells. Dcp1-positive cells were calculated in a sample of *n*=5 for each genotype. (C,F,I,N) The statistical analysis was carried out using an unpaired Student's *t*-test. Data are mean±s.e.m. Statistical significance is indicated in each graph: ***P<0.001; **P<0.01; *P<0.05. All graphs were plotted using GraphPad Prism 8.3.1. All the imaginal discs are oriented posterior to the left and dorsal upwards. All eye-antennal imaginal discs were captured at 20× magnification and adult eyes at 10× magnification, unless specified otherwise. Scale bars: 100 µm.

It is possible that the reduced-eye phenotype of *ey>miR-137* is due to induction of cell death. Therefore, we tested a series of cell death markers in the eye-antennal disc of third-instar larvae. We used Death caspase-1 (Dcp-1) in *Drosophila*, a homolog of mammalian caspase 3, as an apoptotic cell marker to mark developmental cell death. Full-length Dcp-1 does not have proteolytic activity; however, when cleaved Dcp-1 is active, the cells undergo apoptosis (Mehta et al., 2021; Song et al., 1997). In comparison to the control, *ey-*Gal4, eye imaginal disc (Fig. 4A,A′), the *ey>miR-137* background showed an increase in the number of retinal neurons undergoing cell death (Fig. 4B,B′). Statistical analysis of the number of Dcp1-positive cells between *ey*-Gal4 and *ey*>*miR-137* confirmed a significant increase in Dcp-1 positive cells (Fig. 4C). We also employed a *hid*5′F-WT-GFP reporter for pro-apoptotic factor *head involution defective* (*hid*), which is involved in regulating apoptosis and programmed cell death to mark dying cells. The reporter is expressed in cells undergoing apoptosis (Tanaka-Matakatsu et al., 2009). In controls, a basal level of GFP (green) signal that marks *hid*5′F-WT-GFP reporter expression is generally seen in a few cells of the third-instar eye-antennal imaginal disc (Fig. 4D,D′), whereas the number of dying cells marked by the *hid5′*F-WT-GFP reporter was significantly higher in gain-of-function *miR-137* (*ey>miR-137*) eye discs, even though the discs were smaller (Fig. 4E,E′). Statistical analysis of *hid5′* GFP mean signal intensity exhibited a significant increase in the *miR-137* gain-of-function background (*ey>miR-137*) when compared to the control (Fig. 4F).

A TUNEL assay detects fragmented DNA ends and marks the nuclei of the dying cells (White et al., 1994). Using a TUNEL assay, we quantitate the cell death in *ey>miR-137* eye disc. In comparison to the *ey-*Gal4 control eye discs (Fig. 4G,G′), there was a significant increase in the number of dying cells in the *ey>miR-137* eye disc (Fig. 4H,H′). Statistical analysis of TUNEL-positive nuclei mean signal intensity confirmed a significant increase in the *miR-137* gain-of-function background (*ey>miR-137*) when compared to the control (Fig. 4I). To test if the observed reduction in eye size was due to caspase-dependent cell death, we misexpressed P35, which is a pan-caspase inhibitor (Hay et al., 1994). In comparison to controls (*ey>P35*) (Fig. 4J,K), co-expression of *miR-137* and P35 (*ey>miR-137+ P35*) in developing eye significantly rescued the *ey>miR-137* phenotype, as seen in the eye imaginal disc and the adult eye (Fig. 4L,M). Statistical analysis of Dcp-1-positive cells in *ey>P35* and *ey>miR-137+P35* eye imaginal discs revealed a significant reduction in the number of Dcp-1-positive cells in experimental conditions (*ey>miR-137+P35*) with respect to the control (Fig. 4N). Thus, blocking cell death using P35 can rescue the *ey>miR-137* reduced-eye phenotype. However, it is not a complete rescue. Therefore, the reduced-eye phenotype of *ey>miR-137* may not be totally dependent on caspase-dependent cell death mechanisms.

We also tested autophagy, which is another mode of homeostasis involving a cell elimination pathway (Das et al., 2012; Shpilka et al., 2011), using the well-established *Atg8a-*mCherry reporter line (Shukla et al., 2019). We checked the levels of Atg8a as an indicator of the entire process of autophagy in the gain-of-function background of *miR-137*. However, we found no significant difference in the levels of *atg8a*-mCherry between *ey>GFP* control and *ey>miR-137* discs (Fig. S3). We also tested if downregulating autophagy using *Atg8a^1^* mutant can rescue the *miR-137-*mediated reduced-eye phenotype. However, reducing *Atg8a* function using the heterozygous mutant of *Atg8a^1^* in *miR-137* gain-of-function background (*ey>miR-137*+*Atg8a^1^*) did not affect the reduced-eye phenotype (Fig. S3). These data suggest that *miR-137* might increase cell death through apoptosis but not through autophagy. It will be important to investigate if *miR-137* affects other developing fields.

### Gain of function of *miR-137* suppresses the size of other developing fields

**Fig. 5.**
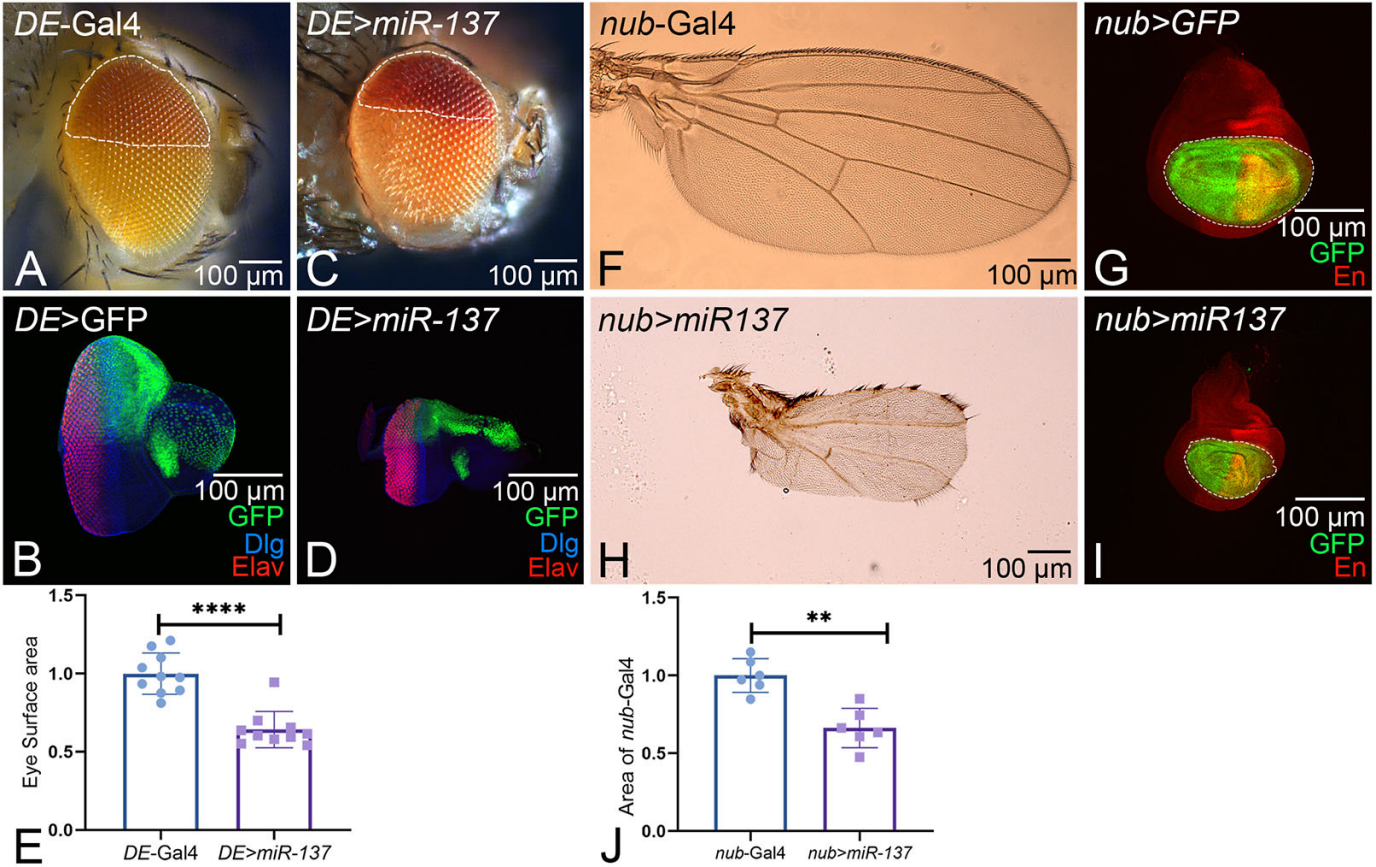
**The gain-of-function phenotype of *miR-137* does not exhibit domain constraint.** (A-D) Gain of function of *miR-137* in the dorsal half of the adult eye (A,C, marked by white dotted lines) using the DE-Gal4 driver (domain marked by GFP reporter) marked by expression of mini-white reporter (red, white dotted line outlines the domain). (C,D) The gain of function of *miR-137* in the background of *DE*-Gal4 *(DE>miR-137*) reduces the dorsal domain of the eye field in (C) the adult eye and (D) the eye-imaginal disc. (B,D) Eye-antennal imaginal disc of third-instar larvae stained for Elav (red), Dlg (blue) and GFP (green) reporter. (E) Dorsal half expression of *DE>miR-137* (*n*=10) normalized to the control *DE*-Gal4 (*n*=10). (E) The bar graph shows the length of the dorsal half of eye in *DE>miR-137* (*n*=10) normalized to the control, *DE*-Gal4 (*n*10). (F,H) Adult wings of (F) *nub*-Gal4 and (H) *nub>miR-137*. The wings of *nub>miR-137* (H) are reduced when compared to the control (F) *nub*-Gal4. (G,I) Wing imaginal discs of (G) *nub*-Gal4 and (I) *nub>miR-137* stained for Engrailed (En) (red, a posterior compartment fate marker) and GFP (green, reporter marks *nub-*Gal4 expression domain). (J) A *nub*-Gal4 expression domain in the wing disc (*n*=5) of *nub>miR-137* normalized to control *nub*-Gal4 (*n*=5). The *nub*-Gal4 domain is significantly reduced in the *nub>miR-137* background. Data are mean±s.e.m. Statistical significance is indicated in each graph: ****P<0.0001; **P<0.01. All graphs were plotted using GraphPad Prism 8.3.1. All eye-antennal imaginal discs were captured at 20× magnification and adult eyes at 10× magnification, unless specified otherwise. Scale bars: 100 µm.

We assayed the effect of the gain of function of *miR-137* in other developing fields to address whether there is any domain-specific constraint in the gain-of-function phenotype of *miR-137* with reference to the compartments within the eye and other developing fields such as the wing. In comparison to the control, in which DE-Gal4 drives expression of the mini-white reporter in the dorsal compartment of the adult eye (Fig. 5A, boundary of the dorsal compartment of the adult eye is marked by dotted white line) and expression of the GFP (green) reporter in the dorsal half of the eye imaginal disc (Fig. 5B), gain of function of *miR-137* using DE-Gal4 (*DE>miR-137*) driver showed a significant reduction in the dorsal compartment of eye imaginal disc and the adult eye (Fig. 5C,D). Furthermore, statistical analysis of the surface area of the reduced-eye phenotype of *DE>miR-137* adult eye showed a significant reduction (∼30%) compared to *DE*-Gal4 control (Fig. 5E). Additionally, the overall size of the eye imaginal disc was reduced because the generation of the DV boundary is crucial for eye development (Singh et al., 2012). Furthermore, we also tested another developing field similar to wing imaginal disc. We employed nub-Gal4 driver to misexpress the gene of interest in the wing pouch region. As compared to the control *nub*-Gal4 adult wing and wing disc (Fig. 5F,G), gain of function of *miR-137* using *nub*-Gal4 (*nub>miR-137*), which drives expression in the wing pouch region, marked by a GFP reporter in the larval wing imaginal disc (Fig. 5G), exhibits a significant reduction of the wing pouch region in the wing disc (Fig. 5I) as well as in the adult wing (Fig. 5H). The statistical analysis of the wing pouch area of *nub>miR-137* wing imaginal disc showed a significant reduction (∼30%) compared to the *nub*-Gal4 control (Fig. 5J). We also tested the gain-of-function phenotype of *miR-137* in the entire fly using *Tub*-Gal4 (*Tub>miR-137*), which caused embryonic or early larval lethality. We also employed a temperature-sensitive Gal80 (Gal80^ts^) approach to titrate the levels of *miR-137* but failed to achieve survival of the adults (data not shown). Overall, our data suggest that *miR-137* affects the size of the developing field.

### *miR-137* targets *Myc* in the developing eye

**Fig. 6.**
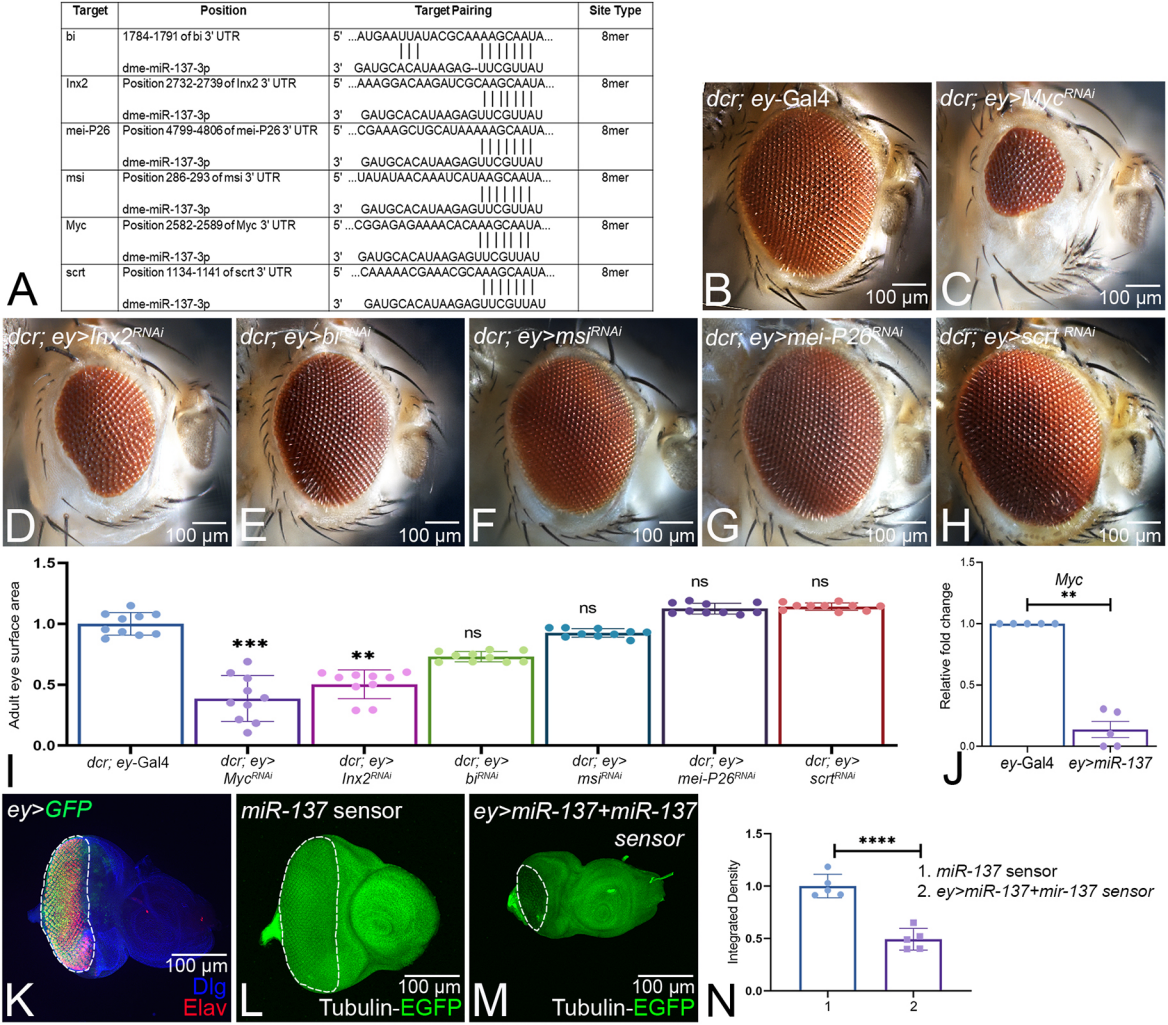
**Screening for *miR-137* gene target(s) identified *Myc*.** (A) The *miR-137* binding site on its target(s) predicted by the bioinformatic tools TargetScan and DIANA. (B-H) Loss-of-function phenotypes in the adult eye of the putative targets identified using bioinformatics approaches. Adult eye phenotypes of (B) *dcr2; ey*-Gal4 (control), (C) *dcr; ey>Myc^RNAi^*, (D) *dcr; ey>Inx2^RNAi^*, (E) *dcr; ey>bi^RNAi^*, (F) *dcr; ey>msi^RNAi^*, (G) *dcr; ey>mei-P26^RNAi^* and (H) *dcr; ey>scrt^RNAi^*. In these experiments, *dcr2* is co-expressed in the *ey* expression domain for a stronger RNA interference effect. (C) *dcr; ey>Myc^RNAi^* and (D) *dcr; ey>Inx2^RNAi^* exhibit a strong reduction in eye size when compared to the control (B). (I) The normalized eye-surface area of *miR-137* targets (*n*=10 per genotype) compared to the control *dcr; ey*-Gal4. The statistical analysis was performed using a one-way ANOVA with Dunnett's multiple comparison test. (J) Relative transcriptional (expression) levels of *Myc* (from triplicate sets) obtained using quantitative PCR (qPCR) in *ey*-Gal4 and *ey>miR-137* backgrounds. Statistical analysis was carried out using an unpaired Student's *t*-test for independent samples. (K) Eye-antennal imaginal disc of third-instar larvae stained for Elav (red), Dlg (blue) a membrane-specific marker and GFP (green) reporter expressing *in ey-*Gal4 domain. (L,M) The *Tubulin–GFP–miR-137* sensor shows ubiquitous expression of GFP under the control of tubulin promoter in the eye discs (L) and the *ey>miR-137+miR-137-sensor* (*ey>miR-137+sensor*) shows reduced GFP fluorescence in *ey*-producing cells the eye discs (M). (N) Quantification of GFP shows a significant reduction in GFP intensity in *ey>miR-137+miR-137-sensor* eye discs when compared to the control. The statistical analysis was carried out using an unpaired Student's *t*-test. Data are mean±s.e.m. Statistical significance is indicated in each graph: ****P<0.0001; ***P<0.001; **P<0.01. All graphs were plotted using GraphPad Prism 8.3.1. All eye-antennal imaginal discs were captured at 20× magnification and adult eyes at 10× magnification unless specified otherwise. Scale bars: 100 µm.

To understand the mechanism underlying the *miR-137*-mediated reduced-eye phenotype, we screened for *miR-137* target genes using bioinformatics analysis tools such as TargetScanFly (http://www.targetscan.org/fly_72/) and DIANA (https://dianalab.e-ce.uth.gr/microt_webserver/#/interactions) to predict the potential targets of *miR-137* (Maragkakis et al., 2009; Tastsoglou et al., 2023) (Fig. 6A). TargetScanFly predicts the targets of fly miRNA by matching the seed sequence (consensus sequence) of the 3′ untranslated region of the miRNA and their target mRNA. miRNAs bind the 3′UTR of their target mRNAs and thereby regulate gene expression. We found multiple putative targets predicted by bioinformatics analysis, including *Myc, Innexin 2* (*Inx2*), *bifid* (*bi*)*, musashi* (*msi*) and *meiotic-P26* (*mei-P26*) (Fig. 6A). To identify the *miR-137* target(s), we used genetic approaches. The rationale was that the loss-of-function phenotype of the target gene(s) in the developing eye should be similar to the gain of function of *miR-137* (*ey>miR-137*). We used the *ey*-Gal4 driver to downregulate expression of the putative targets using RNAi lines to determine if they exhibit a reduced-eye phenotype (Fig. 6B-H). In these experiments, co-expression of *dcr2* with a RNAi transgene results in a stronger phenotype by enhancing the RNA interference effect. In comparison to the control, *dcr; ey*-Gal4 (Fig. 6B), downregulation of *Myc* (*dcr; ey>Myc^RNAi^*) (Fig. 6C) and *Inx2 (dcr; ey>Inx2^RNAi^*) (Fig. 6D) produces a reduced-eye phenotype*.* However, *bi (dcr; ey>bi^RNAi^*) (Fig. 6E), *msi* (*dcr; ey>msi^RNAi^*) (Fig. 6F), *mei-p26 (dcr; ey>mei-P26^RNAi^*) (Fig. 6G) and *scratch* (*scrt*; *dcr; ey>scrt^RNAi^*) (Fig. 6H) did not show any reduction in eye size. The eye surface area of the *miR-137* targets was quantified, and the statistical analyses revealed that *Myc* and *Inx2* could be the targets, as their downregulation results in a reduced-eye phenotype similar to that in *ey>miR-137* (Fig. 6I). However, the frequency of rescue of the reduced-eye phenotype with *Inx2* was not as high as with *Myc* (data not shown). We used reverse transcription-quantitative PCR (qPCR) to determine the expression levels of *Myc*, the target of *miR-137,* in the eye imaginal disc. We found a significant reduction in *Myc* expression levels (∼70%) in the gain-of-function background of *miR-137* (*ey>miR-137*) as compared to the control (Fig. 6J). Thus, we confirmed *Myc* as the target of *miR-137*, the absence of which in the developing eye affects the eye size.

To further validate that *miR-137* targets *Myc* in the developing eye and that the *ey>miR-137*-induced reduced-eye phenotype is due to downregulation of the target *Myc*, we generated the *Tubulin–GFP–miR-137* sensor line (Brennecke et al., 2003). We modified the Tubulin-GFP-sensor toolbox with the 3′ UTR of *Myc* (which has a *miR-137*-binding site). In the control eye discs, GFP is ubiquitously expressed in all the cells (Fig. 6L). In the *ey>miR-137* background, *miR-137* mRNA will bind to its 3′ UTR binding site on *Myc*, and will degrade the mRNA with GFP and the *miR-137*-binding site. This will reduce the GFP levels in ey-Gal4 driver domain (Fig. 6K) where *miR-137* is misexpressed. This reduction in GFP levels in the *ey*-Gal4 driver expression domain will serve as evidence that *Myc* is the target of *miR-137*. The larval eye discs in the *Tubulin–GFP–miR-137* sensor flies expressing *miR-137* exhibited reduced GFP levels in the *ey*-expressing cells (Fig. 6M, highlighted by a white dotted line) when compared to the *Tubulin–GFP–miR-137* sensor controls (Fig. 6L). Quantification of GFP levels in the eye discs expressing *Tubulin–GFP–miR-137* showed a significant reduction of GFP signal intensity levels in the *Tubulin–GFP–miR-137* sensor larvae expressing *miR-137* in the *ey* cells when compared to the control discs (Fig. 6L). Taken together, these findings strongly suggest that *Myc* is the target of *miR-137*.

### Modulation of *Myc* levels affects the reduced-eye phenotype of *miR-137* gain of function

**Fig. 7.**
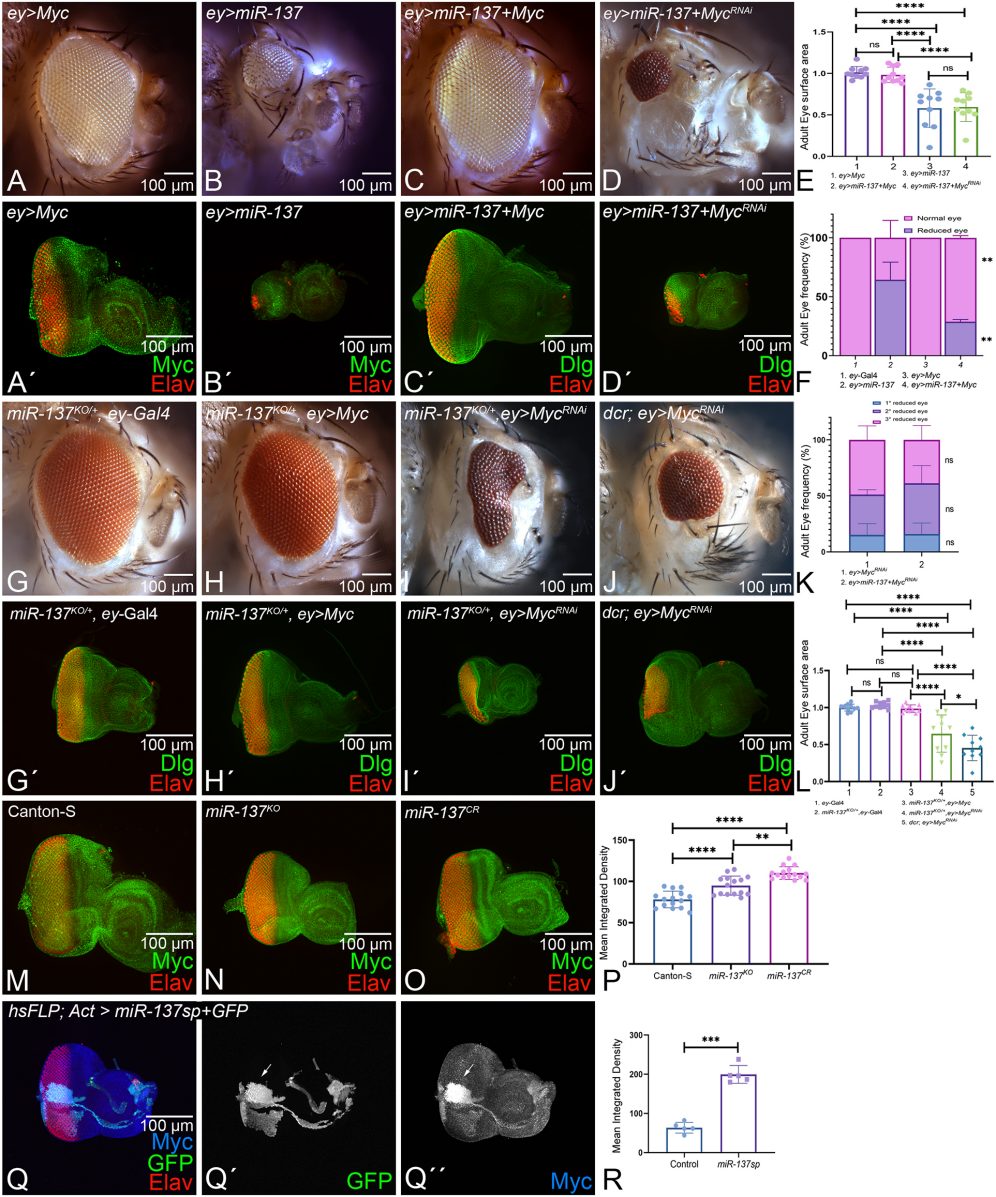
***miR-137* targets *Myc* during eye development.** (A-B′) Gain of function of *Myc* (*ey>Myc*) (A,A′) and *miR-137 (ey>miR-137)* (B,B′) in (A,B) adult eye and (A′,B′) eye imaginal disc. (C′,D′) Eye-antennal imaginal discs stained for Elav, a pan-neuronal marker (red), and Dlg, a membrane-specific marker (green). (C-D′) Modulation of *Myc* levels in *ey>miR-137* background using (C,C′) gain of function of *Myc* (*ey>miR-137+Myc)* and (D,D′) loss of function of *Myc* (*ey>miR-137+Myc^RNAi^*), as seen in (C,D) adult eye and (C′,D′) eye imaginal disc. (E) The normalized eye-surface area of (A) *ey>Myc*, (B) *ey>miR-137*, (C) *ey>miR-137+Myc* and (D) *ey>miR-137+Myc^RNAi^* showed a significant increase in eye size in *ey>miR-137 +Myc* compared to controls (*ey>miR-137*, *n*=10 per genotype). (F) Statistical analysis of adult eye phenotype frequency between (B) *ey*>*miR-137* and (C) *ey>miR-137+ Myc*. There was significant rescue seen in *ey>miR-137+ Myc* when compared to *ey>miR-137.* (G-H′) Loss of function of *miR-137* in (G,G′) *ey*-Gal4 (*miR-137^KO^*, *ey*-Gal4) and (H,H′) *ey>Myc* (*miR-137^KO^*, *ey*>*Myc*) background produces a wild-type phenotype in (G,H) adult eye and (G′,H′) eye imaginal disc. (I-J′) Loss of function of *Myc* in (I,I′) *miR-137^KO^* (*ey>miR-137^KO^+Myc^RNAi^)* and (J,J′) *ey*-Gal4 (*ey>Myc^RNAi^*) background, as seen in (I,J) adult eye and (I′,J′) eye imaginal disc, produces a reduced-eye phenotype. (K) Statistical analysis of adult eye phenotype frequency in (J) *ey*>*Myc^RNAi^* and (D) *ey>miR-137+Myc^RNAi^*. The reduced-eye phenotype is classified into: (1) 1° reduced eye, representing reduced eye in one or both of the eyes (blue); (2) 2° reduced eye, representing highly reduced to no eye in one of the eyes (purple); and (3) 3° reduced eye, representing highly reduced to no eye in both the eyes (pink). (L) The normalized eye-surface area of *ey-*Gal4 (Fig. 1B,C), *miR-137^KO/+^, ey-*Gal4 (G,G′), *miR-137^KO/+^, ey>Myc* (H,H′), *miR-137^KO/+^, ey>Myc^RNAi^* (I,I′) and *dcr*; *ey>Myc^RNAi^* (J,J′). (M-O) Eye-antennal imaginal disc of (M) Canton-S, (N) *miR-137^KO^* and (O) *miR-137^CR^* stained for Elav (red) and Myc (green). (P) Mean integrated density of *Myc* levels from Canton-S (M), *miR-137^KO^* (N) and *miR-137^CR^* (O). The signal intensity of Myc was calculated in five regions of interest per disc within a sample of *n*=5 for each genotype. Myc intensity was significantly increased in *miR-137^KO^* and *miR-137^CR^*. (Q-Q″) Eye-antennal imaginal disc of (Q) random flp out clones of *miR-137sp (hsFLP; Act>miR-137sp+ GFP*) stained for Elav (red) and Myc (blue). (Q′) Single channel image of GFP marking the clones of *miR-137sp*. (Q′′) Single channel image of Myc. Myc intensity was dramatically increased with flp out clones of *miR-137sp*. (R) Mean integrated density of *Myc* levels between control and flp out clones of *miR-137sp.* The signal intensity of Myc was calculated at the ROI (50×50) of flp out clones (*n*=5) and non-flp out (*n*=5) of the same disc. Myc intensity was significantly increased in hsFLP-out clones of *miR-137sp*. One-way ANOVA with Tukey's multiple comparison test was used for comparing more than two groups, and an unpaired Student's *t*-test was used to compare two groups. For adult frequency analysis, three replicates of 200 flies (200×3=600) were used to calculate the frequency of each genotype. Data are mean±s.e.m. Statistical significance is indicated in each graph: ****P<0.0001; ***P<0.001; **P<0.01; *P<0.05. All eye-antennal imaginal discs were captured at 20× magnification and adult eyes at 10× magnification unless specified otherwise. Scale bars: 100 µm.

*Myc* is a regulator of growth during development (Gallant et al., 1996; Prober and Edgar, 2002). To determine if *miR-137* has a role in growth regulation like its target *Myc*, we employed classical genetic approaches. The rationale was that if *Myc* is the downstream target of *miR-137*, then gain of function of *Myc* can rescue the reduced-eye phenotype of *ey>miR-137*. Conversely, loss of function of *Myc* will result in the reduced-eye phenotype (*ey>Myc^RNAi^*). In comparison to the control, *ey*-Gal4 (Fig. 1B,D), gain of function of *Myc* (*ey>Myc*) exhibits near wild-type eye (Fig. 7A,A′), and gain of function of *miR-137* (*ey>miR-137*), which served as control, shows the reduced-eye phenotype in the adult eye and eye imaginal disc (Fig. 7B,B′). In comparison to the controls, gain of function of *miR-137* and *Myc* together (*ey>miR-137+ Myc)* results in rescue of the reduced-eye phenotype of *ey>miR-137* alone, as seen in the adult eye and the eye imaginal disc (Fig. 7C,C′). Conversely, downregulation of *Myc* using a RNAi strategy in the *ey>miR-137* background (*ey>miR-137+Myc^RNAi^*) results in a reduced-eye phenotype (Fig. 7D,D′). The statistical analysis of adult eye-surface area analysis showed that gain of function of *Myc* in the *ey>miR-137* background (*ey>miR-137+ Myc*) rescues the reduced-eye phenotype significantly (Fig. 7E). Furthermore, gain of function of *Myc* in the *ey>miR-137* background (*ey>miR-137+ Myc*) significantly reduced the frequency of the reduced-eye phenotype of the gain of function of *miR-137* (*ey>miR-137,* Fig. 7F). Additionally, gain of function of *miR-137* along with downregulation of *Myc ^RNA^*^i^ (*ey>miR-137+Myc^RNAi^*) exhibit the reduced-eye phenotype, as seen in controls such as *ey>miR-137* and *ey> Myc^RNAi^* alone. However, the frequency of the reduced-eye phenotype did not change significantly, suggesting that it is a threshold-dependent response (Fig. 7K).

Conversely, we also modulated *Myc* levels in the heterozygous background of *miR-137^KO^/+* (knockout, downregulated miR-137 levels due to heterozygosity) (Fig. 7G-I). Here, *miR-137^KO^/+*, which exhibits near-normal eyes, served as a control (Fig. 7G,G′). Gain of function of *Myc* in the *miR-137^KO^/+* background (*miR-137^KO^/+*; *ey>Myc*) exhibits a normal eye (Fig. 7H,H′), whereas downregulation of *Myc* using a RNAi approach in the *miR-137^KO^/+* background (*miR-137^KO^/+*; *ey>Myc*^RNAi^) exhibits the reduced-eye phenotype (Fig. 7I,I′). The control *ey>Myc^RNAi^* also exhibits a reduced-eye phenotype (Fig. 7J,J′), which is similar to gain of function of *ey>miR-137* in the eye imaginal disc and adult eye (Fig. 7B,B′). The statistical analysis of adult eye surface areas of these different combinations revealed that there is no significant change in eye size when Myc levels are upregulated in *miR-137^KO^/+* background. However, there is a significant reduction in eye size when Myc levels are downregulated in the *miR-137^KO^/+* background (Fig. 7L). These results suggest that modulation of *Myc* expression levels can change the *ey>miR-137* eye phenotypes. Thus, *Myc* is one of the downstream targets of *miR-137* in the developing eye.

Additionally, we used Canton-S (Fig. 7M) to identify changes in Myc levels in *miR-137^KO^* (Fig. 7N) and *miR-137^CR^* (Fig. 7O). The statistical analysis of mean integrated density of Myc showed a significant increase in Myc levels in *miR-137^KO^* and *miR-137^CR^* backgrounds (Fig. 7P). Furthermore, random FLP-out clones of *miR-137sponge* (*miR-137sp*) were created using heat-shock Flippase in developing eye imaginal discs (Fig. 7Q), where GFP marks the clones of FLP-out *miR-137sp* (*hsFLP; Actin>y+>GAL4/UAS-miR-137sp*). These *miR-137sp* clones showed a robust increase in Myc levels (Fig. 7Q′,Q″, marked by white arrows). The statistical analysis of mean integrated density of Myc (Fig. 7R) showed a significant increase in Myc levels in random FLP-out clones of *miR-137sp*. These results demonstrate that Myc expression levels are significantly increased in *miR-137^KO^* and *miR-137^CR^*, and in random FLP-out clones of *miR-137sp*, emphasizing the role of *miR-137* in targeting *Myc* to regulate its expression in developing eye.

### *miR-137* targets *Myc* expression for the growth regulation response

**Fig. 8.**
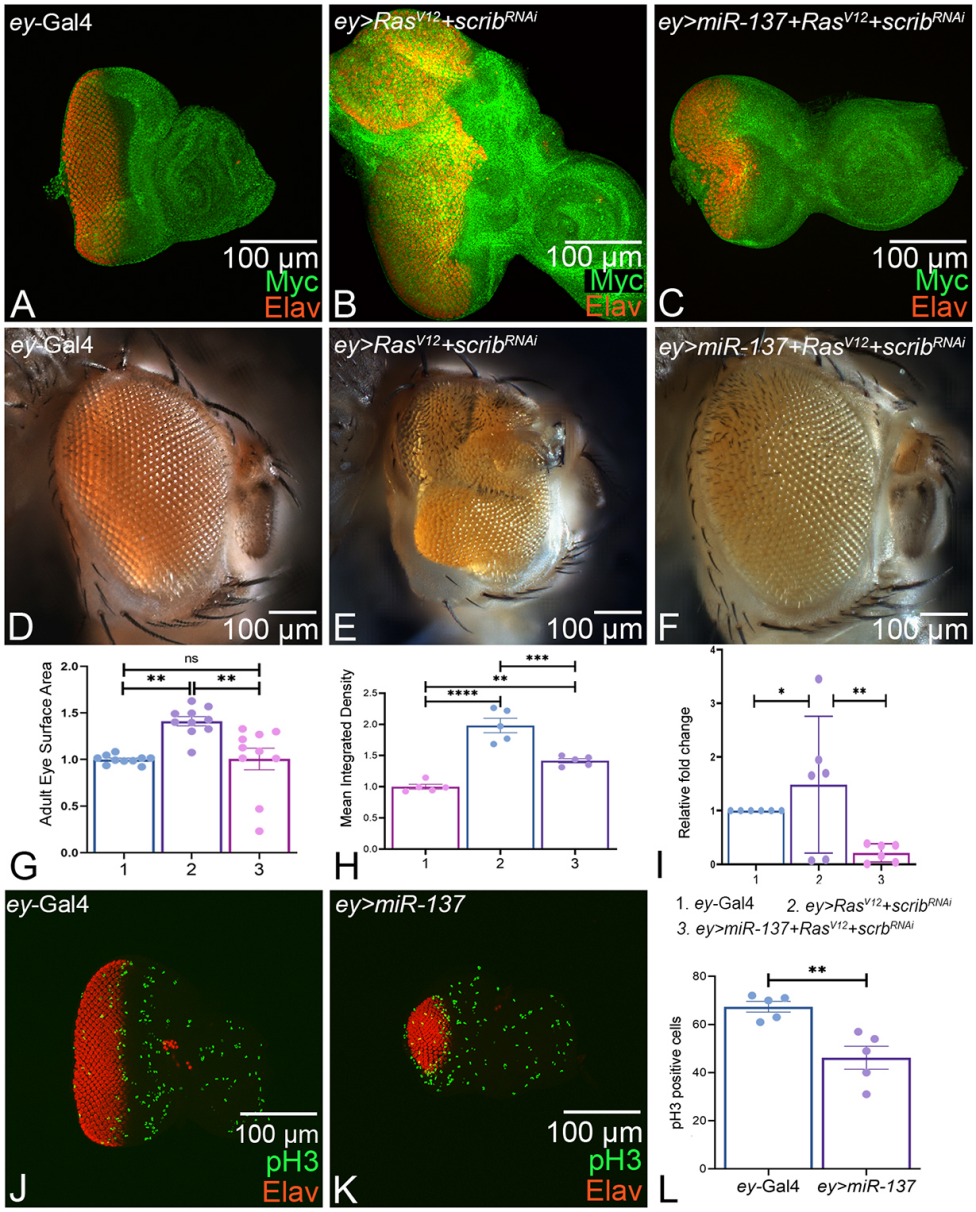
***miR-137* exhibits growth regulation function in the developing eye.** (A,D) Eye imaginal disc (A) and adult eye (D) of *ey-*Gal4 control. (B,E) *ey>Ras^V12^+ scrib^RNAi^* serves as a model for oncogenic cooperation resulting in neoplastic tumors and overgrowth. (C,F) Gain of function of *miR-137: ey>miR-137+ Ras^V12^+ scrib^RNAi^* significantly rescues the overgrowth phenotype of (B,E) *ey> Ras^V12^+ scrib^RNAi^* both at the eye imaginal disc level and in the adult eye. (A-C) All eye-imaginal discs are stained for *Myc* (green) and Elav (red). (G) Statistical analyses of normalized adult eye surface area (pixels/inch) were carried out using one-way ANOVA with Tukey's multiple comparison test for (A) *ey-*Gal4, (C) *ey> Ras^V12^+ scrib^RNAi^* and (E) *ey> miR-137+Ras^V12^+ scrib^RNAi^* (*n*=10 per genotype). The eye surface is reduced by gain of function of *miR-137* in the *ey> Ras^V12^+ scrib^RNAi^* background. (H) Average intensity of *Myc* levels within the three backgrounds: (1) *ey-*Gal4, (2) *ey> Ras^V12^+ scrib^RNAi^* and (3) *ey>miR-137+ Ras^V12^+ scrib^RNAi^*. The signal intensity of *Myc* was calculated in three region of interest per disc within a sample of *n*=5 for each genotype. Statistical analysis was performed using an unpaired Student's *t*-test for independent samples. *Myc* intensity was significantly reduced in the *ey>miR-137+ Ras^V12^+ scrib^RNAi^* background. (I) Relative transcriptional (expression) levels of *Myc* using the quantitative real-time PCR (qRT-PCR) in *ey*-Gal4, *ey>Ras^V12^+scrib^RNAi^* and *ey> Ras^V12^+scrib^RNAi^+miR-137* backgrounds. Statistical analysis was carried out in triplicate, using an unpaired Student's *t*-test for independent samples. *Myc* transcript levels were significantly increased in *ey>Ras^V12^+scrib^RNAi^* compared to *ey*-Gal4 and decreased in *ey> Ras^V12^+scrib^RNAi^*+*miR-137* background compared to *ey*-Gal4. (J,K) Eye-antennal imaginal disc of (J) *ey*-Gal4 and (K) *ey>miR-137* stained for phospho-histone 3 (pH3) (green, a marker for cell division) and Elav (red). (L) Statistical analysis of pH3 counts in the retinal field marked by Elav in comparable region of interest in *ey*-Gal4 (*n*=5) and *ey>miR-137* (*n*=5) shows a significant difference in proliferating cells between control and *ey>miR-137*. The statistical analysis was carried out using an unpaired Student's *t*-test. Quantification of pH3 and eye-surface area was calculated using Fiji/ImageJ software to assay the differences. Data are mean±s.e.m. Statistical significance is indicated in each graph: ****P<0.0001; ***P<0.001; **P<0.01; *P<0.05. All graphs were plotted using GraphPad Prism 8.3.1. All the imaginal discs are oriented posterior to the left and dorsal upwards. All eye-antennal imaginal discs were captured at 20× magnification and adult eyes at 10× magnification unless specified otherwise. Scale bars: 100 µm.

During tumorigenesis, Myc and Ras are the two most commonly activated oncogenes. *Myc* is upregulated in tumor models (Atkins et al., 2016; Prober and Edgar, 2002). Since *Myc* is one of the targets of *miR-137*, we tested the role of *miR-137* in regulating *Myc* expression levels in a well-established oncogenic cooperation model of *Ras^V12^; scrib^RNAi^*. Gain of function of Ras along with downregulation of *scrib* using a RNAi approach (*ey>Ras^V12^+scrib^RNAi^*) result in neoplastic tumors (Brumby and Richardson, 2003; Pagliarini and Xu, 2003). In comparison to the *ey*-Gal4 control (Fig. 8A,D,G), there is a significant increase in the eye size in the *ey>Ras^V12^*+*scrib^RNAi^* (Fig. 8B,D,G) background, along with high levels of *Myc* signal intensity (Fig. 8H). Gain of function of *miR-137* in the oncogenic cooperation model (*ey>miR137+ Ras^V12^+ scrib^RNAi^)* resulted in a significant reduction of the overgrowth phenotype of *ey> Ras^V12^*+*scrib^RNAi^* in eye imaginal disc and the adult eye (Fig. 8C,F), which was further validated by statistical analysis of surface area comparisons (Fig. 8G), by the Myc staining signal intensity (Fig. 8H) and by the adult eye phenotypes frequency (Fig. S4). Gain of function of *miR-137* in the *ey>Ras^V12^+scrib ^RNAi^* background (*ey>miR-137 +Ras^V12^+scrib^RNAi^*) (Fig. 8C,F,G) results in a significant reduction in *Myc* levels (Fig. 8H). We also compared the relative transcriptional (expression) levels of the *Myc* mRNA in the developing eye using a qPCR approach. We found that *Myc* transcript levels were significantly increased in *ey>Ras^V12^+scrib^RNAi^* compared to *ey*-Gal4 and were decreased in the *ey> Ras^V12^+scrib^RNAi^*+*miR-137* background compared to *ey*-Gal4 (Fig. 8I).

To understand whether the reduced-eye phenotype of *ey>miR-137* is due to decreased cell proliferation, we assessed expression of anti-phospho-histone H3 (pH3), which is a mitotic marker. In the histone H3 tail, phosphorylation of a highly conserved serine residue (Ser-10) marks the onset of mitosis and represents the completion of the cell cycle (Kim et al., 2017). The gain of function of *miR-137* (*ey>miR-137*) exhibits reduced cell proliferation (Fig. 8K) as compared to the *ey*-Gal4 control (Fig. 8J). In the control eye disc, these cells can be seen as a band of cells at the MF and in a broad domain, anterior to the MF, in the head capsule region (Fig. 8J). These results suggest that the reduction in the eye field in the *ey>miR-137* background may also be due to reduced cell proliferation rates. Statistical analysis of pH3 counts in the retinal field marked by Elav in a comparable region of interest in *ey*-Gal4 and *ey>miR-137* revealed a significant difference in proliferating cells between control and *ey>miR-137*, suggesting that *miR-137* also affects cell proliferation.

### *miR-137* targets Myc to regulate growth and patterning of the developing eye field

**Fig. 9.**
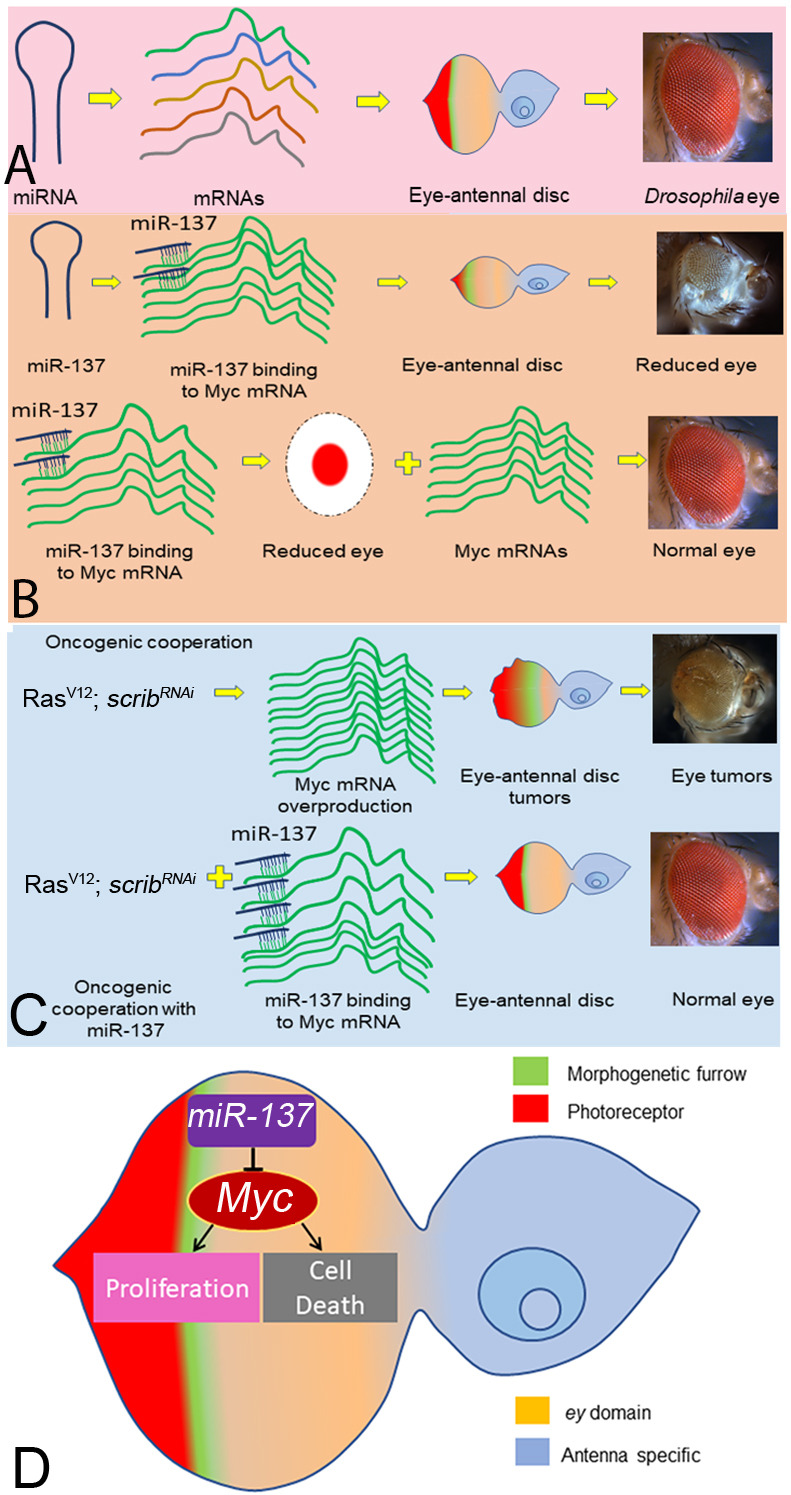
**A model to demonstrate the role of *miR-137* in growth regulation by targeting *Myc* during eye development.** (A) When miRNAs are expressed at normal levels, a normal eye is produced. (B) Gain of function of *miR-137* in the *ey*-Gal4 domain (*ey>miR-137*) results in a reduced-eye phenotype. Our results indicate that *miR-137* targets *Myc* to reduce the size of eye. (C) *miR-137* targets *Myc* to regulate growth during development. Gain of function of *miR-137* can rescue overgrowth phenotypes observed in the tumor model of oncogenic cooperation in *Drosophila*. (D) Our results show that *miR-137* targets *Myc* to regulate cell proliferation and cell death in the developing eye and in other tissues.

Based on all these data, we provide a model with which we demonstrate the growth regulation function of *miR-137*, a microRNA, which binds to 3′UTR of *Myc* mRNA to generate double-stranded RNA to induce *Myc* mRNA degradation (Fig. 9A,B). Consequently, *Myc* levels are downregulated, which results in a reduced-eye phenotype. Thus, *miR-137* regulates the levels of its downstream target *Myc*, which in turn regulates both cell death and cell proliferation to maintain the size of the developing eye field (Fig. 9).

## DISCUSSION

In eukaryotes, microRNAs (miRNAs) represent a class of small non-coding RNAs (∼22 nucleotides in length) that post-transcriptionally regulate protein-coding gene expression by translational repression or by RNA degradation of target mRNA. The human genome comprises a number of miRNA genes that account for nearly 1-5% of all predicted human genes that regulate ∼30% of all protein-coding genes (Shang et al., 2023; Vishnoi and Rani, 2023). It has been estimated that every miRNA can interact with an average of 200 mRNA transcripts (Colaianni and De Pittà, 2022). Consequently, miRNAs may play a fundamental role in biological processes such as development, metabolism, proliferation, growth regulation, apoptotic cell death and diseases. The structural and genetic similarities between the visual system of *Drosophila* and vertebrates, along with evolutionary conservation of miRNAs involved in development and regulation, underscore the value of the fruit fly as a model organism. These similarities suggest that *Drosophila*, with its strong repertoire of molecular genetic tools, can provide valuable insights into the molecular and neuronal circuits regulated by miRNAs in the specification and functioning of the visual system, which is relevant to so-called higher organisms, such as humans (Bier, 2005; Deshpande et al., 2024; Shang et al., 2023; Singh and Irvine, 2012). *Drosophila* has a genome-wide collection of miRNA overexpression and miRNA depletion transgenes (Fulga et al., 2015). We employed the *Drosophila* eye model for a forward genetic screen to look for the miRNAs involved in patterning and growth (Deshpande et al., 2024, 2023). We identified *miR-137* as a negative regulator of the eye because the gain of function of *miR-137* results in the reduced-eye phenotype due to activation of negative regulators of eye development, which results in MF progression. Our qPCR data, along with the high-throughput miRNA data (Leader et al., 2018), show that *miR-137* is expressed during the development, particularly in early development. Interestingly, the *miR-137* gain-of-function phenotype is similar in the wing and in another developing field, which suggest that *miR-137* gain of function exhibits a phenotype of reduction in size of a developing field, as seen in the eye and wing. However, the gain-of-function phenotype of the entire fly larva could not be observed due to early lethality.

### *miR-137* employs caspase-dependent cell death

Gain of function of *miR-137* can activate cell death, which results in the reduced-eye phenotype. However, when we block the caspase-dependent cell death pathway using the baculovirus P35 (Hay et al., 1994), there was rescue of the reduced-eye phenotype by *miR-137* gain of function in a small population of flies. Since the rescue was not complete and the frequency of rescue was less, it suggests that other mechanisms might be involved. A basal level of cell death occurs in the developing eye to maintain cellular homeostasis. During development, both caspase-dependent cell death, called apoptosis, and a lysosomal-mediated degradation process, called autophagy, are used to regulate patterning and growth (Chimata et al., 2023). These data rule out the possibility of any significant role of autophagy in the reduced-eye phenotype of *miR-137* gain of function (Fig. S3). However, in certain conditions, *Myc* promotes autophagy (Nagy et al., 2013). Therefore, *miR-137*-mediated reduced-eye phenotype employs caspase-dependent cell death.

### *miR-137* targets *Myc* to regulate growth

We screened for the target of *miR-137* using bioinformatics, followed by molecular-genetic analysis, and found that loss of function of *Myc* and *Inx2* using a RNAi strategy produced reduced-eyes phenotypes. However, the extent and frequency of the phenotype of *Myc* was highly consistent. *Myc*, an oncogene and a bHLH class transcription factor, is the master regulator, controlling diverse processes such as homeostasis. In *Drosophila, Myc* mutants have smaller eyes and body size (Johnston et al., 1999). Furthermore, complete loss of *Myc* (null mutant) results in larval lethality (Pierce et al., 2004), which is similar to the gain of function of *miR-137* in the entire larva using the *TubP*-Gal4 driver. Since *Myc* is upregulated in the majority of cancers, it is plausible that our findings, where *Myc* is identified as a target of *miR-137*, the gain of function of which produces the reduced-eye phenotype, are significant. We further validated *Myc* as a target for *miR-137* using a qPCR assay, which showed that *Myc* levels are significantly reduced in the gain-of-function background of *miR-137* in the developing eye. Furthermore, the reduced-eye phenotype produced by the gain of function of *miR-137* was rescued by the gain of function of *Myc*. Thus, *Myc* is a target of *miR-137* in the developing eye.

Our studies suggest that optimum levels of *miR-137* are crucial for development, as gain of function of *miR-137* (*ey>miR-137*) produces a reduced-eye phenotype, whereas loss of function causes the converse phenotype of an enlarged eye. Interestingly, we found that gain of function of *miR-13*7 ectopically induced Wg expression, whereas *Myc* levels are downregulated as *Myc* is its target. Interestingly, both *Myc* and *wg*, which is a secretory morphogen, regulate growth. Wg represses Myc levels in the zone of non-proliferating cells (ZNC) at the *Drosophila* wing margin. Blocking Wg signaling with dominant-negative TCF restores Myc expression (Gallant, 2013; Johnston et al., 1999). Our findings suggest *miR-137* plays a role in this repressive interaction between Wg and Myc during development.

### *miR-137* regulates *Myc* in *Drosophila* tumor models

During eye development, *Myc* prevents ommatidium loss and eye size reduction (Huang et al., 2017), and its mutation leads to smaller eyes and body size (Johnston et al., 1999). We have shown a reduced-eye phenotype in both *Myc^RNAi^* and *ey>miR-137*, where the phenotype of the latter emulates the former. The oncogenic cooperation between Ras^V12^ and *scrib^RNAi^* drives malignant tumors in the eye-antennal disc, although individually, these constructs can only drive benign tumors. *Myc* plays a central role in the oncogenic cooperation between *Ras^V12^* and *scrib^RNAi^* (Atkins et al., 2016), where increased *Myc* levels promote hyperplasia in *Ras^V12^* (Prober and Edgar, 2002) and *scrib^RNAi^*. We have shown that *miR-137* reduces tumor growth in *Ras^V12^* and *scrib^RNAi^* by targeting *Myc*, supporting a tumor-suppressor role for *miR-137*. Further studies will uncover the molecular mechanisms by which *miR-137* regulates eye development, and the expression of *Myc* and other target genes (Fig. S4).

### *miR-137* is conserved and implicated in diseases

Based on the miRbase release 22 (https://mirbase.org/), *D. melanogaster* currently consists of 471 mature and 238 precursor miRNA(s), while *Homo sapiens* consists of 2693 mature and 1917 precursor miRNA(s) (Ambros et al., 2003; Kozomara and Griffiths-Jones, 2014). Most miRNA(s) belong to highly conserved non-coding sequences, where conservation of mature sequences is common (Price et al., 2011). Based on the conserved seed sequence, the *Homo sapiens* (human) ortholog of *D. melanogaster miR-137* is *hsa-miR-137*. Furthermore, the *Drosophila miR-137* shows an 87% similarity in the mature sequence to humans (Ibáñez-Ventoso et al., 2008) (Fig. S5). Recent genome-wide association studies (GWASs) have shown that single-nucleotide polymorphisms near *miR-137* are statistically associated with neurodegenerative and psychiatric disorders (Pergola et al., 2024; Whalley et al., 2012). They regulate neural stem cell proliferation and differentiation during development (Sun et al., 2011), and their host gene, *MIR-137*HG, is associated with many psychiatric disorders (Whalley et al., 2012). Furthermore, Myc overexpression is linked to immune disorders such as myasthenia gravis, psoriasis and pemphigus vulgaris.

The *Drosophila miR-137* human homolog *hsa-miR-137*, which is located on chromosome 1p22, acts as a tumor suppressor in colorectal cancer, squamous cell carcinoma and melanoma (Rupaimoole and Slack, 2017). In many cancers, *miR-137* inhibits proliferation and differentiation (Althoff et al., 2013; Bi et al., 2018; Silber et al., 2008), which has led to its recognition as a therapeutic target in glioma (Wang et al., 2020). It has been reported that high levels of *Myc* expression in many cancers are associated with poor survival and disease progression. In many cancers, *miR-137* can serve as tumor suppressor by targeting the mRNAs that encode oncoproteins and thereby inhibit proliferation and differentiation (Althoff et al., 2013; Bi et al., 2018; Silber et al., 2008). Around 70% of human cancers showed deregulation of *Myc* (Sorolla et al., 2020), and has been suggested as a target for molecular therapeutics (Duffy et al., 2021; Sorolla et al., 2020; Wang et al., 2021). Both *miR-137* and Myc have been identified as possible therapeutic targets, but no studies have reported *Myc* as a direct target of *miR-137* in humans and *Drosophila.* Interestingly, in a cohort of 706 patients with high KRAS expression and SCRIB loss, 395 patients (56%) exhibited low copy number expression (log2≤−0.12) of *miR-137/5p,* suggesting a significant heterozygous loss that may further affect the tumor-suppressive activity of *miR-137* (Cerami et al., 2012; de Bruijn et al., 2023; Gao et al., 2013). These studies highlight the utility of *miR-137* as a possible therapeutic target for cancers linked to *Myc* overexpression. Recently, another study reported that protein tyrosine phosphatase 61F (PTP61F), and ortholog of mammalian TC-PTP/PTP18, is one of the targets of *miR-137*, which is a highly conserved brain-enriched miRNA (Saedi et al., 2024). PTP61F is known to dephosphorylate the Insulin receptor InR (InR-P). These studies demonstrate that miR-137 regulates levels of PTP61F to maintain normal insulin signaling and energy homeostasis. In the future, it will be interesting to study molecular-genetic interactions between targets of *miR-137*, such as Myc and PTP61F, which are involved in growth regulation and tissue homeostasis.

### Study limitations

microRNA characterization studies have some challenges regarding redundancy and conservation of expression amongst species, both of which may affect their targets and functions. This is because miRNA families often share common seed sequences, leading to functional redundancy. Findings from tissue-specific expression of miRNAs from one model can be extrapolated to another only after verification of expression.

## MATERIALS AND METHODS

### Fly stocks

The fly stocks used in this study are described in FlyBase (https://flybase.org/). We have used *ey*-Gal4 (Hazelett et al., 1998) and UAS-*miR-137* (BL59881) to screen the eye phenotype described. Other stocks used include UAS-*bi^RNAi^* (BL28341), UAS-*E(z)^RNAi^* (BL31617), UAS-*Inx2^RNAi^* (BL29306), UAS-*mei-P26*^RNAi^ (BL36855), UAS-*mitf* ^RNAi^ (BL34835), UAS-*msi^RNAi^* (BL55152), UAS-*Myc^RNAi^* (BL25783) and UAS-*scrt^RNAi^* (BL27025) to screen for the targets of *miR-137*. Additionally, miR*-137^KO^* (Chen et al., 2014), *miR*-137CR (Saedi et al., 2024), UAS-*miR-137* sponge (*miR-137sp*, BL61395), UAS-Myc (BL9764) (Johnston et al., 1999), *hid5′FWT-GFP* (Tanaka-Matakatsu et al., 2009), UAS-*P35* (Hay et al., 1994), UAS-mCherry-*Atg8a* and *Atg*8a mutant were used (Shukla et al., 2019). A stock UAS-*Ras^V12^*; UAS-*scrib^RNAi^*; was used to study the neoplastic tumors generated using UAS-*scrib^RNAi^* (BL59080) and *UASRas^V12^* (BL64196) (Snigdha et al., 2021). In this study, we have used *ey*-Gal4 driver (Hazelett et al., 1998), *eyg*-Gal4 (Jang et al., 2003), DE-Gal4 (Morrison and Halder, 2010), *nubbin-*Gal4 (Verghese et al., 2012), TubP (Tubulin)-Gal4 (BL5138) and TubP-Gal80^ts^; Tub-Gal4/TM6B (BL67065) for characterizing the role of *miR-137* in eye, wing and whole fly development. All candidate RNAi lines were co-expressed with UAS-*dicer2* (BL24650) by crossing them to the Gal4 driver(s) to increase the efficacy of RNA interference effects (Dietzl et al., 2007). All the stocks are maintained at 25°C on cornmeal, yeast and molasses food medium.

### Generation of Tubulin-GFP-SV40 sensor for *Myc*

We have modified the original *Tubulin–GFP–SV40* sensor, gifted by Eric Lai (Sloan-Kettering Institute, New York, USA), with the addition of tubulin promoter sites. We generated the *miR-137* sensor line by inserting the miR-137 binding sites of 3′UTR of *Myc* into the *tubulin-GFP-SV40* plasmid (gifted by Eric Lai, Sloan-Kettering Institute, New York, USA) between the NotI and XhoI restriction sites (GenScript). Transgenic *Drosophila* strains were generated by standard injection with the Δ2-3 helper transposase (GenetiVision) (Brennecke et al., 2003) using random P element insertion and balanced to perform experiments.

### Genetic crosses

The Gal4/UAS system was used to misexpress the transgene(s) of interest in a spatiotemporal manner (Brand and Perrimon, 1993). All crosses were maintained at 25°C and 29°C, unless specified, to observe the changes in induction levels (Singh et al., 2005a).

### Genetic mosaic analysis

To generate gain-of-function random flp out clones in the developing eye, we crossed *y,w, hsFLP^122^; P(Act>y^+^>Gal4)25P (UAS-GFP^S65T^)/CyO* (Struhl and Basler, 1993) flies to the UAS-*miR-137* (gain of function) or *UAS-miR137 sp* (loss of function) flies. The progeny from these cultures were heat-shocked to generate flp out clones.

### Immunohistochemistry

Wandering third-instar larvae were dissected for the eye-antennal imaginal disc in 1×phosphate-buffered saline (PBS), fixed in 4% paraformaldehyde (fixative) in 1×PBS for 20 min and washed thrice in PBS-Triton-X100 (PBST). The tissues (eye-imaginal discs) were blocked using the serum and stained using a combination of antibodies following the standard protocol (Singh et al., 2002; Tare et al., 2020). Primary antibodies used were mouse anti*-*β-GAL (1:100; Developmental Studies Hybridoma Bank, DSHB), goat anti-Atonal (Ato; 1:250; Santa Cruz Biotechnology, sc-15699), goat anti-Homothorax (Hth; 1:250; Santa Cruz Biotechnology, sc-26187), mouse anti-Dachshund (Dac; 1:100; DSHB), rabbit anti-Death Caspase-1 (Dcp1; 1:250; Cell Signaling Technology, 9578), mouse anti-Disc-large (Dlg; 1:100; DSHB), rat anti- Embryonic Lethal Abnormal Vision (Elav; 1:100; DSHB), mouse anti-Eyes absent (Eya; 1:100; DSHB), rabbit anti-Myc (1:100; Santa Cruz Biotechnology, D2704), mouse anti-Phospho histone 3 (pH3; 1:200; Cell Signaling Technology, 9701), mouse anti-Scabrous (Sca; 1:100; DSHB), mouse anti-Prospero (Pros; 1:100; DSHB) and mouse anti-Wingless (Wg; 1:100; DSHB). The discs were washed thrice in PBST for 10 min. Secondary antibodies used were donkey anti-rat IgG conjugated to Cy5 (1:250; 712-175-153), donkey anti-rabbit IgG conjugated to Cy3 (1:300; 711-166-152) or goat anti-mouse IgG conjugated to Cy3 (1:200; 115-166-062) (all from Jackson ImmunoResearch). Discs were mounted in Vectashield and scanned on a Fluoview 3000 Laser Scanning Confocal Microscope (Steffensmeier et al., 2013; Tare et al., 2016). We captured the images at 20× magnification unless specified. We prepared and analyzed the figures using Adobe Photoshop CS6 software.

### Detection of cell death

Cell death was detected by using a Terminal Transferase dUTP Nick End Labeling (TUNEL) kit from Roche Diagnostics. A standardized protocol was used for TUNEL staining in the eye antennal imaginal discs (Chimata et al., 2022; White et al., 1994). The TUNEL-positive cells were counted from five sets of imaginal discs per genotype. These numbers were used for statistical analysis the *P-*values were calculated using an unpaired Student's *t*-test (Deshpande et al., 2024, 2023). The graphs were plotted using GraphPad Prism 8. The error bars represent the s.e.m. *P<0.05, **P<0.01, ***P<0.001.

### Adult eye imaging

The adult flies were frozen at −20°C for ∼4 h before imaging their eyes. The flies were mounted onto the needles after dissecting the wings and legs, and imaged at 10× magnification. The needle with the fly is inserted in a clay putty on a glass slide to position it stably for the lateral view of the fly eye and head. Images were taken on a MrC5 color camera mounted on an Axioimager.Z1 Zeiss Apotome using a *z*-sectioning function of Axiovision software 4.6.3 (Cutler et al., 2015; Gogia et al., 2020b; Irwin et al., 2020; Mehta et al., 2021). The final *z*-projections were compiled using Adobe Photoshop CS6 software.

### Frequency of eye phenotype

Three independent sets of 200 flies were screened (200×3=600) and the frequencies of eye phenotype(s) were calculated for each genetic cross (Raj et al., 2020; Singh et al., 2024). The eye phenotypes were categorized as reduced-eye or normal eye. The graphs were plotted using statistical methods described below in GraphPad Prism 8.

### Quantitative analyses of eye surface area and eye imaginal discs

Image J software was used to quantify the area (pixels/inch) as described previously (Deshpande et al., 2021). We measured the eye surface area by drawing the region of interest (ROI) along the perimeter of the adult eye. The values were analyzed and plotted as mean±s.e.m. using the statistical methods described below. Each genotype was analyzed with at least ten images, unless specified otherwise. For measuring intensity in eye imaginal discs, three ROIs of 50×50 pixels were selected and normalized to controls (Deshpande et al., 2021). Each genotype was analyzed with at least five images unless specified otherwise.

### Adult wing imaging

The adult flies were sequentially immersed in 50% and 70% of ethanol for 2 days, and 90% of ethanol for 4 days. Wings were then dissected in 70-100% ethanol and mounted using Canada balsam. Images were taken on a MrC5 color camera mounted on an Axioimager.Z1 Zeiss Apotome using a *z*-sectioning function of Axiovision software 4.6.3. The final *z*-projections were compiled using Adobe Photoshop CS6 software.

### Real-time quantitative PCR

We used 60-80 eye-antennal imaginal discs for RNA isolation (Mehta and Singh, 2017; Mehta et al., 2019). The tissue was collected and homogenized in TRIzol reagent (Invitrogen, 15596026). We used RNA Clean & Concentrator (Zymo Research, R1013) columns after total RNA extraction and eluted in about 20 µl DNase/RNase-free water. The quality and quantity of RNA were assayed by a Nanodrop 2000 spectrophotometer (Thermo Fisher Scientific). The cDNA was synthesized from 500 ng of total mRNA through a reverse transcription reaction using a first-strand cDNA synthesis kit (GE Healthcare, 27926101). Real time-qPCR was performed using BioRad iQ SYBR Green Supermix (Bio-Rad, 1708860) according to the standard protocol (Chimata et al., 2023; Deshpande et al., 2024, 2023). For miRNA gene expression levels, miRNA isolation from 60-70 pairs of eye-antennal imaginal discs was executed using a miRNeasy kit (Qiagen, 217084). The reverse transcription was performed using a TaqMan MicroRNA Reverse Transcription Kit (ThermoFisher Scientific, 4366596) with *miR-137* and 2S-rRNA probes (ThermoFisher Scientific, 4440886 and 4427975). Real time-qPCR was performed using a TaqMan Fast Advances Master Mix (ThermoFisher Scientific, 4444557) with probes (*miR-137* and 2S-rRNA). Fold change was calculated using the comparative CT method (2^−ΔΔCT^ method). The primers used were: GAPDH-Fwd, 5′-GCGGATAAAGTAAATGTGTGC-3′; GAPDH-Rev, 5′-AGCTCCTCGTAGACGAACAT-3′; *Myc*-Fwd, 5′-TGTCCTCGATGTGCTCAACC-3′; *Myc*-Rev, 5′-CACTATCAGAGCCGGTCGTC-3′; *miR-137*, 5′-UAUUGCUUGAGAAUACACGUAG-3′; 2S rRNA, 5′-TGCTTGGACTACATATGGTTGAGGGTTGTA-3′.

### Statistics

We used GraphPad Prism 8 for all our statistical analysis: two-way ANOVA with Sidak multiple comparison tests and an unpaired Student's *t*-test for the eye frequency data; a non-parametric one-way ANOVA, Mann–Whitney test for mean integrated intensity quantification data (>2 groups). Data are plotted as mean±s.e.m. with the individual values indicated. Statistical significance is set at 95% CI and is shown by ****P<0.0001, ***P<0.001, **P<0.01 and *P<0.05 (Chimata et al., 2023).

## Supplementary Material

10.1242/develop.204373_sup1 Supplementary information
