# Supplementary material for: miR-137 targets Myc to regulate growth during eye development
Source: Development. 2025 Jul 16;152(14):dev204373. doi: 10.1242/dev.204373 (PMC12338917; doi:10.1242/dev.204373)
Supplement: Supplementary information [file develop-152-204373-s1.pdf]

Supplementary Figure 1

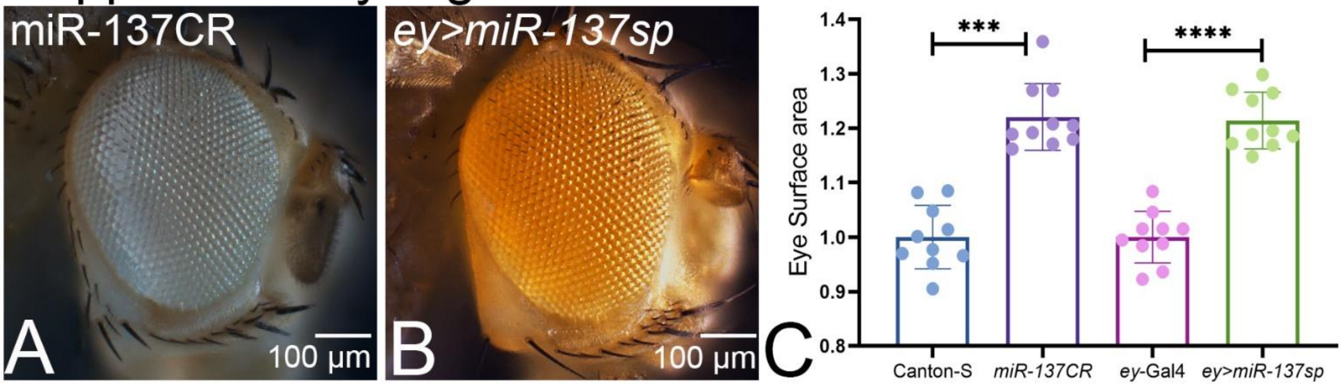

Fig. S1. Loss-of-function of *miR-137* results in enlarged eye

Adult eye of (A) miR-137CR-KO (B) *ey>miR-137 sponge* (C) Statistical analysis of normalized adult eye surface area (pixels/inch) calculated between Canton-S and miR-137CR-KO, and ey-Gal4 and *ey>miR-137 sponge* (n=10, per genotype). Graph representing quantification of adult eye surface area (µm^2) using Fiji/ImageJ software (NIH) with statistical analysis of normalized adult eye surface area (pixels/inch) using with One-way ANOVA with Sidak's multiple comparison test. All graphs were plotted using GraphPad Prism 8.3.1. Error bars represent standard error of mean (mean ± SEM). Statistical significance in each graph is shown by *p*-value: ****$p<0.0001$, ***$p<0.001$; **$p<0.01$; *$p<0.05$; ns- non-significant. Adult eyes at 10X magnification unless specified. Scale bar = 100 µm.

Development • Supplementary information

Supplementary Figure 2

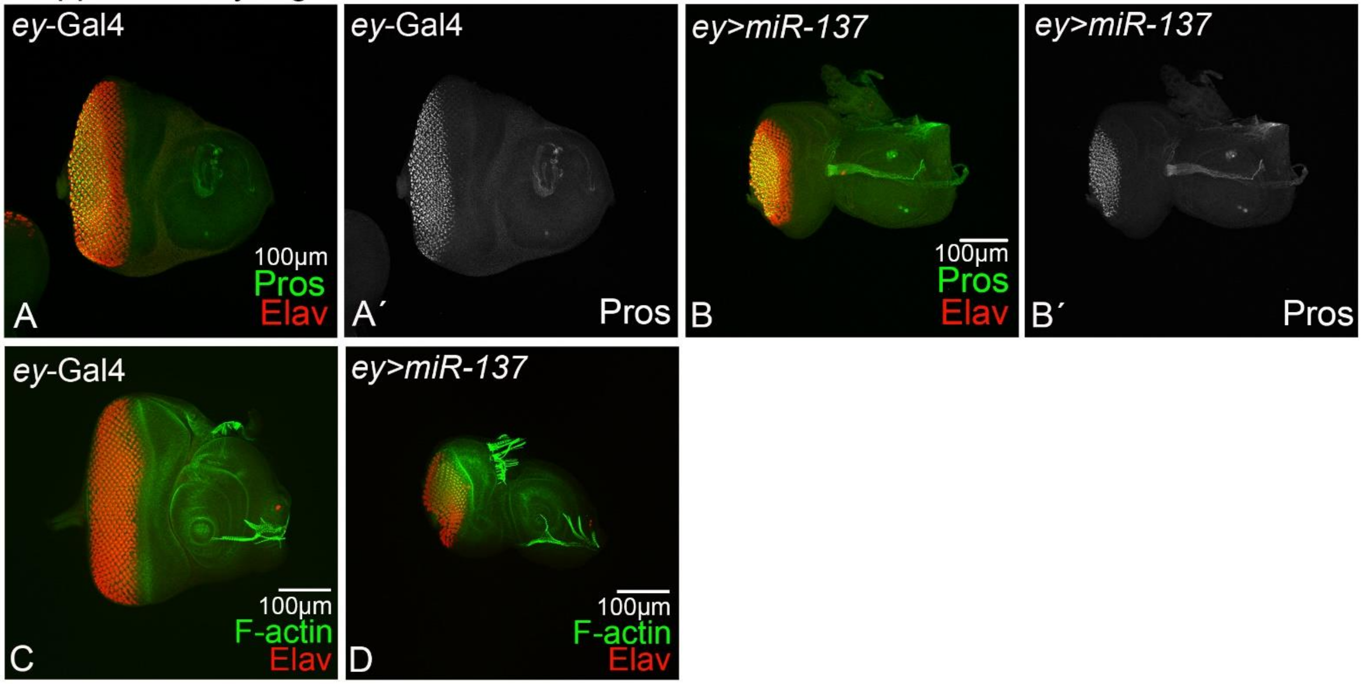

Fig. S2. Gain-of-function of miR-137 affects retinal differentiation marker and morphogenetic furrow (MF) movement

Eye-antennal imaginal disc stained for retinal differentiation genes (A, B) Pros (green) and Elav (red) in eye-antennal imaginal disc of (A) *ey*-Gal4 control and (B) *ey>miR-137*. Split channel for (A′, B′) Pros. Note that in *ey>miR-137* Pros expression domain has reduced, compared to *ey*-Gal4 control. Eye-antennal imaginal disc stained for (C, D) F-actin (green) and Elav (red) in eye-antennal imaginal disc of (C) *ey*-Gal4 control and (D) *ey>miR-137*. Note that in *ey>miR-137*, MF movement is perturbed, compared to *ey*-Gal4 control. All the imaginal discs are oriented posterior to the left and dorsal up. The magnification of all eye-antennal imaginal discs is at 20X unless specified. Scale bar = 100 μm.

Development • Supplementary information

Supplementary Figure 3

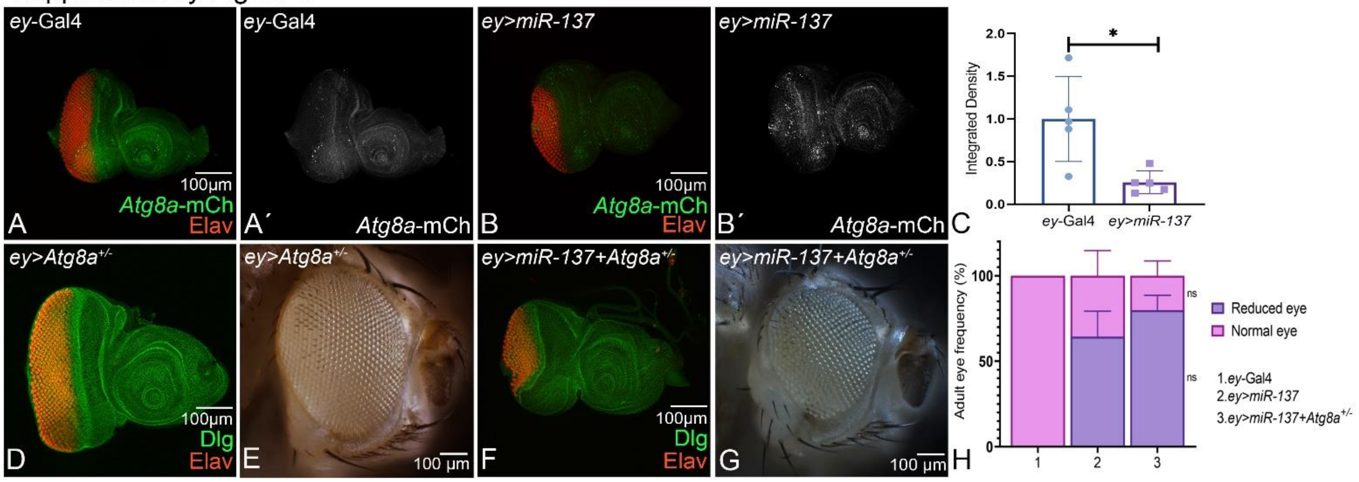

Fig. S3. Autophagy mediated cellular homeostasis is not responsible for reduced-eye phenotype of miR-137 gain-of-function in developing eye.

Eye-antennal imaginal disc stained for *Atg8a*-mCherry (A, B) *Atg8a*-mCherry (green) and Elav (red) in eye-antennal imaginal disc of (A) *ey*-Gal4 control and (B) *ey>miR-137*. Split channel for (A′, B′) *Atg8a*-mCherry. Note that in *ey>miR-137, Atg8a*-mCherry reduced, compared to *ey*-Gal4 control. (C) Graph representing quantification of mCherry integrated density (µm^2) using Fiji/ImageJ software (NIH) with statistical analysis of unpaired student’s t-test. Eye-antennal imaginal disc stained for Dlg (D, F) Dlg (green) and Elav (red) in eye-antennal imaginal disc of (D) *ey*-Gal4 control and (F) *ey>miR-137*. Note that *ey>miR-137* showed reduced photoreceptors, compared to *ey*-Gal4 control. Adult eye of (E) *ey>Atg8a$^{+/-}$* control (G) *ey>miR-137+ Atg8a$^{+/-}$* (H) Graphical representation of adult eye phenotype frequency of indicated genotypes (n=200, 3 replicates) was performed using the Two-way ANOVA with Sidak’s multiple comparison test. All graphs were plotted using GraphPad Prism 8.3.1. Error bars represent standard error of mean (mean ± SEM). Statistical significance in each graph is shown by *p*-value: ****$p<0.0001$, ***$p<0.001$; **$p<0.01$; *$p<0.05$; ns- non-significant. Orientation of all the imaginal discs is posterior to the left and dorsal up. All eye-antennal imaginal discs are imaged at 20X magnification, and adult eyes at 10X magnification unless specified. Scale bar = 100 µm.

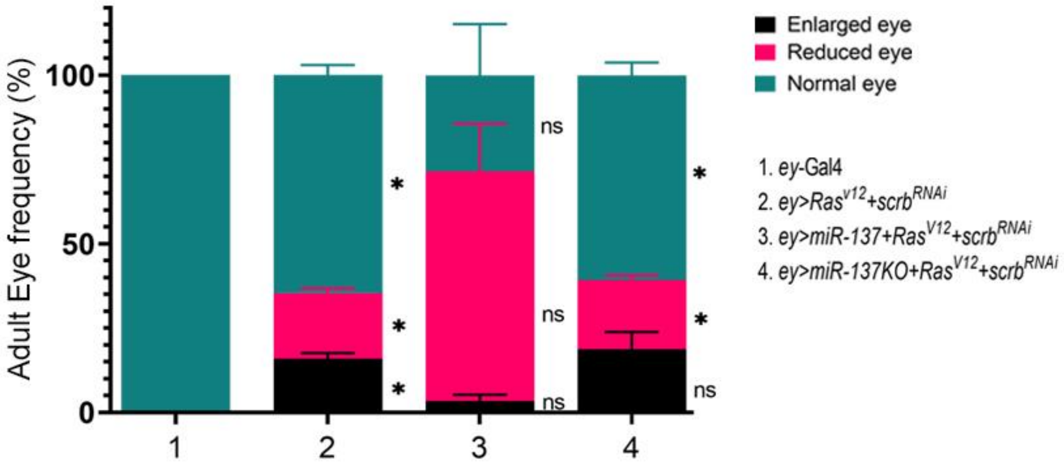

Fig. S4. Graphical representation of adult eye phenotype frequency of *ey*-Gal4 control, *ey>Ras*^{V12} +*scrb*^{RNAi}, *ey>miR-137+Ras*^{V12}*+scrb*^{RNAi} and *ey>miR-137KO+Ras*^{V12}*+scrb*^{RNAi} (n=200, 3 replicates) was performed using Two-way ANOVA with Tukey's multiple comparison test. All graphs were plotted using GraphPad Prism 8.3.1. Error bars represent standard error of mean (mean ± SEM). Statistical significance in each graph is shown by *p*-value: ****p<0.0001, ***p<0.001; **p<0.01; *p<0.05; ns- non-significant.

Supplementary Figure 5

```
#CLUSTAL O

hsa-miR-137-3p      UUAUUGCUUAAGAAUACGCGUAG    23
dme-miR-137-3p      -UAUUGCUUGAGAAUACACGUAG    22
                     ******** ******* *****

#Results for hsa-miR-137-3p  vs dme-miR-137-3p :
  Alignment length: 23
Identical residues: 20
  Similar residues: 0
  Percent identity: 86.96
Percent similarity: 86.96
```

Fig. S5. Clustal analysis shows that *Drosophila miR-137* is conserved. *Drosophila miR-137* shows an 87% similarity in the mature sequences to humans *has-miR-137*.
